# RETRACTED ARTICLE: Enhanced glycemic control, pancreas protective, antioxidant and hepatoprotective effects by umbelliferon-α-D-glucopyranosyl-(2^I^ → 1^II^)-α-D-glucopyranoside in streptozotocin induced diabetic rats

**DOI:** 10.1186/2193-1801-2-639

**Published:** 2013-11-28

**Authors:** Vikas Kumar, Danish Ahmed, Firoz Anwar, Mohammed Ali, Mohd Mujeeb

**Affiliations:** 1 Department of Pharmaceutical Sciences, Faculty of Health Sciences, Sam Higginbottom Institute of Agriculture, Technology & Sciences, Allahabad, Uttar Pradesh 211007 India; 2 Sidharatha Institute of Pharmacy, Dehradun, Uttrakhand 248001 India; 3 Department of Phytochemisty & Pharmacognosy, Faculty of Pharmacy, Jamia Hamdard, New Delhi 110062 India

**Keywords:** Umbelliferon-α-D-glucopyranosyl-(2I → 1II)-α-D-glucopyranoside, Streptozotocin, Antidiabetic, Antihyperlipidemic, Glibenclamide

## Abstract

**Objective:**

The objective of the present study was to evaluate the effect of umbelliferon-α-D-glucopyranosyl-(2I → 1II)-α-D-glucopyranoside (UFD) from *Aegle marmelos* Corr. on serum glucose, lipid profile and free radical scavenging activity in normal and STZ (streptozotocin) induced diabetic rats.

**Materials and methods:**

Diabetes was induced by single interperitoneal injecting of streptozotocin (60 mg/kg, i.p.) in the rats. All the rats were divided into following groups; I - nondiabeteic, II - nondiabetic + UFD (40 mg/kg, p.o.), III - diabetic control, IV - UFD (10 mg/kg, p.o.), V - UFD (20 mg/kg, p.o.), VI - UFD (40 mg/kg) and VII - glibenclamide (10 mg/kg, p.o.). Serum glucose level and body weight were determined periodically. Biochemical parameter, antioxidant enzyme and histopathology study were performed on the day 28. Oral glucose tolerance test study was performed to identify the glucose utilization capacity.

**Results:**

All the doses of UFD and glibenclamide decrease the level of serum glucose, glycated hemoglobin, glucose-6-phosphatase, fructose-1-6-biphosphate and increased the level of plasma insulin, hexokinase. The UFD doses also showed effects on antioxidant enzymes viz. superoxide dismutase, catalase and glutathione peroxidase which were significantly increased and the level of malonaldehyde was markedly decreased. Histologically study, focal necrosis, deposition of fats, increased the size of the intercalated disc were observed in the diabetic rat liver, kidney, heart and pancreas but was less obvious in treated groups. The mechanism of action of the UFD emerges to be due to increase the activity of antioxidant enzyme and secretion of pancreatic insulin.

**Conclusion:**

Reduction in the FBG (fasting blood glucose), glycated hemoglobin, glucose-6-phosphatase, fructose-1-6-biphosphate, superoxide dismutase, catalase, glutathione peroxides, cholesterol, triglyceride, LDL, VLDL levels and improvement in the level of the plasma insulin, hexokinase, HDL was observed by the UFD treated rats. The result indicates that UFD has anti-diabetic activity along with anti hyperlipidemic and antioxidant efficacy and provides a scientific rationale to be used as an Anti-diabetic agent.

**Electronic supplementary material:**

The online version of this article (doi:10.1186/2193-1801-2-639) contains supplementary material, which is available to authorized users.

## Introduction

Diabetes mellitus (DM) is a group of syndrome characterized by dietary intake, changing in the lifestyle, excessive use of lipid, carbohydrate and protein. Poorly controlled blood glucose level is the major factor in the development of both diabetic complication such as type 1 diabetes and type 2 diabetes (American Association of Diabetes Educators 2002). STZ is mainly used for induction of experimental autoimmune diabetes. Low dose administration of STZ in the peritoneal cavity of an animal is the best model for type I diabetes. Oral hypoglycaemic agents (insulin, sulphonylureas, thiazolidiones and bioguanides) and different plant based drugs were used for the treatment of diabetes, but oral hypoglycaemic drug having some limitation in the treatment of diabetes (Valiathan 1998). The plants based drugs are gaining popularity day by day. These plant based drugs possess active ingredient and act on variety of targets by various mode and mechanism. Several species of plants have been reported in the reputed alternative system of medicine as best choice for the treatment of diabetes because plant based antidiabetic drug are considered less toxic and free from side effects. The major drawback of the natural therapy is limitation of bioactive compound for claiming their antidiabetic effect (Morin 1987). Most of the researchers claimed that diabetes complications were occurred by oxidative stress (Halliwell and Gutteridge 1989). Clinical and experimental condition of diabetes increasing the level of oxidative stress otherwise changes in antioxidant capacity and produced the etiology of chronic diabetes (Ravi et al. 2004). Coumarins widely consumed in the human diet in the form of vegetable and fruits (Hoult and Paya 1996), coumarins present in the food and vegetable play an important role as dietary antioxidants. Many investigator claim that several phenolic coumarins might play a role as dietary antioxidants, because several fruit and vegetable were consumed by human beings as food.

**Figure 1:**
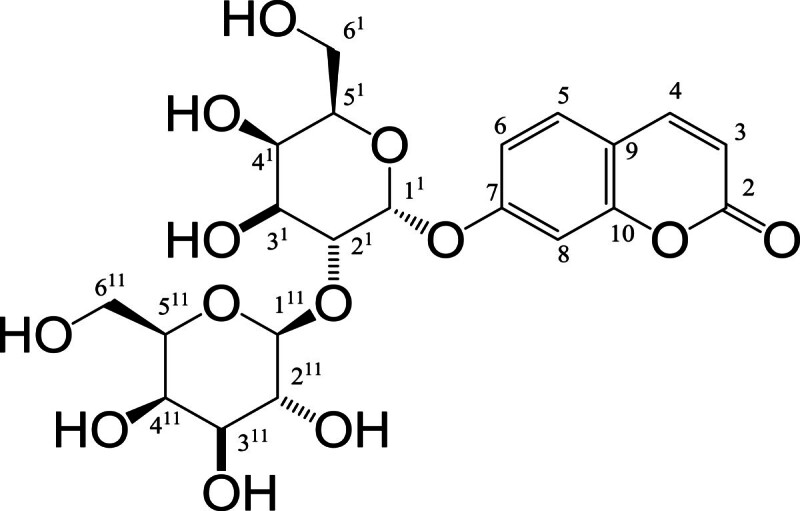
Structure of UFD.

*Aegle marmelos* Corr. (Rutaceae) is a very common plant found especially in hills of the Himalaya, dry forest and south India with altitude (250–1200 m) (Hajra et al. 1997; Gupta and Tandon 2004). Different parts (leaves, fruit, bark and stem) of the plant are used as ethanomedicine against fevers, abdomen pain, palpitation of the heart, urinary troubles, melancholia, anorexia, dyspepsia, diabetes and diarrhea (Badam et al. 2004; Gupta and Tandon 2004). More than 100 chemical constituent were isolated from the *Aegle marmelos* Correa including eugenol, lupeol, aegeline, marmasinin, marmin, skimmianine, aegelin, lupeol, cineole, citral, citronellal, cuminaldehyde (4-isopropylbenzaldehyde), eugenol, marmesinin, marmelosin, luvangetin, aurapten, psoralen, marmelide, fagarine, and tannins. These chemical constituents have been proved active against various disease like malaria, gastrointestinal and cancer disease. Different solvent extracts showed effectiveness against antiulcer, antidiabetic, antioxidant, antihyperlipidemic, antipyretic, anti-inflammatory on various models of animal. But the bioactive compound present in this extract was not identified in their natural process. Presently, there is no published source for the claim about the antidiabetic, antihyperlipidemic and antioxidant activities of bioactive compounds isolated from *A. marmelos* (Litchfield and Wilcoxon 1949; Maity et al. 2009). Umbelliferon-α-D-glucopyranosyl-(2I → 1II)-α-D-glucopyranoside (shown in Figure 1), a glucosidic derivative of coumarin (6-hydroxycoumarin), isolated from the stem bark of *A. marmelos* is powerful antioxidant. The present study investigates the effect of oral administration of bioactive compound, umbelliferon-α-D-glucopyranosyl-(2^I^ → 1^II^)-α-D-glucopyranoside on antidiabetic, antihyperlipidemic and antioxidant effect on STZ-induced diabetic rats.

## Material and methods

### General

Veego, Model No. MPI melting point apparatus was used for melting point. ^1^H NMR spectra were recorded on Bruker Advance II 400 NMR Spectrophotometer and ^13^C NMR spectra on Bruker Advance II 100 NMR Spectrophotometer in DMSO using TMS as internal standard. Mass spectra were obtained on the VG-AUTOSPEC spectrometer. UV λmax (DMSO) were recorded on Shimadzu UV-1700 and FT-IR (in 2.0 cm-1, flat, smooth, Abex) were taken on Perkin Elmer – Spectrum RX-I spectrophotometer.

### Chemical

Streptozotocin (Sigma Chemical Co. USA), GOD/POD kit, Cholesterol kit, Triglyceride kit, (Span, India), Glibenclamide (Ranbaxy, India), Carboxyl methyl cellulose (CMC) (SD fine, India) were purchased from respective vendor. Silica gel (60–120 mesh) (Nicholas India Pvt. Ltd) was used for column chromatography. The entire reagent utilized for experimental protocol and chromatographic isolation were of analytical grade and used without further purification.

### Material

The stem bark of *Aegle marmelos* Correa. was collected from the Botanical Garden, Department of Pharmaceutical Sciences, Faculty of Health Sciences, Sam Higginbottom Institute of Agriculture, Technology & Sciences – Deemed University, Allahabad, Uttar Pradesh, India and authenticated by Dr. Imran Kajmi (Pharmacognosist). A specimen voucher (SIP/HD/054/12) of the plant sample respectively had been deposited in the herbarium of Siddharatha Institute of Pharmacy, Dehradun, Uttarakhand, India.

### Extraction and isolation

The shade dry stem bark of *Aegle marmelos* Correa (2 kg) was extracted with methanol (5 L) at 45°C for 72 hr. After extraction total filtrate was concentrated to dryness in rotatory vacuum evaporator at 40°C to obtain uniform slurry (322 gm) (Kumar et al. 2009; Kumar et al. 2011a; Kumar et al. 2013a). The slurry was dissolved in small amount of methanol and absorbed on silica gel (60–120 mesh). It is subjected to column using as a C_6_H_14_/CHCl_3_/MeOH gradient system (1:0:0, 2:0:0, 4:0:0, 4:1:0, 1:1:0, 1:4:0, 1:6:0, 0:1:0, 0:48:0, 0:24:1, 0:48:2, 0:10:0, 0:10:1, 0:24:7, and 0:47:10; 3.0 L for each gradient system), yielding 22 fractions. The collected fractions spotted on pre coated silica gel TLC plate and the fractions having the same R_f_ value pooled together in 7 fractions. Fraction 8–14 (13.5 g) were combined separated on a silica gel column (CHCl_3_/MeOH, 30:1), and rechromatographed on a silica gel column (CHCl_3_/MeOH, 8:1), yielding 3 subtractions. Compound was separated by a normal phase silica gel column (CHCl_3_/MeOH, 1:4). The compound was found to be 100% pure by HPTLC by using solvent system CHCl_3_/MeOH (20:1), see Figure 1.

### Drugs solution

UFD and glibenclamide were emulsified with 2% carboxyl methyl cellulose (CMC) dissolved in distilled water. Streptozotocin was dissolved in freshly prepared citrate buffer (pH = 4. 5).

### Animals

Male albino rat (Wistar strain 150-200 g) was used for the experiment. The animals were housed under standard conditions of temperature (25 ± 1°C), relative humidity (55 ± 10%), 12 hr/12 hour light/dark cycles and fed on standard pellet diet (Lipton rat feed, Ltd., Pune) and water *ad libitum*. The experimental protocol was approved by the Institutional Animal Ethical Committee of Siddhartha Institute of Pharmacy (1435/PO/a/11/CPCSEA).

### Acute toxicity study

The toxicity studies were adopted as per OESD Guideline No.420; (Annexure-2d) of CPCSEA. For acute toxicity studies in normal healthy rats fasted overnight and randomly divided into five groups and each group contain rats (n = 10). Rats were treated with starting doses (0.05, 0.10, 0.50 and 0.100 g/kg body weight) of test compound and the control group was treated with vehicle alone (CMC 2%; 1 ml/kg body weight). All the animal groups allowed for food and water *ad libitum* and were followed over a period of 2 h for changing in various economical (Defecation and urination), neurological (Spontaneous activities, reactivity, touch response, pain response and gait) and behavior (Alertness, restlessness, irritability, and fearfulness) responses (Litchfield and Wilcoxon 1949; Lipnick et al. 1995). The mortality caused by the extract within this period of the time was observed.

### Assessment of compound in an oral glucose tolerance test (Bonner-weir 1988)

Healthy rats were divided into five groups of six animals each,

Group I (Control): treated with vehicle only.

Group II (UFD): treated with compound 10 mg/kg.

Group III (UFD): treated with compound 20 mg/kg.

Group IV (UFD): treated with compound 40 mg/kg.

Group V (Standard): treated with glibenclamide 10 mg/kg.

All group animals received drug and vehicle orally. After 30 min treatment with different doses of UFD and glibenclamide, all groups rat received 2 gm/kg of glucose. The blood sample collected from the retro-orbit of the eye of rats at regular interval of 0, 30, 60, 90, 120 and 150 min each for their glucose tolerance.

### Induction of diabetes

Diabetes was induced in the Wistar rats by using the single interperitoneal injection of streptozotocin (60 mg/kg body weight). Volume of (STZ) 1 ml/kg body weight prepared by STZ dissolving in freshly prepared 0.01 M citrate buffer (pH = 4.5) (Brosky and Logothelopoulos 1969; Ahmed et al. 2013). After 3 day of STZ administration, blood glucose level of rats was estimated. Rats with a blood glucose level of 270 mg/dL beyond were considered as diabetic.

### Experimental design and schedule

The rats were randomly divided into 7 groups and each group contains 6 animals.

Group I (Normal Control): Untreated group

Group II (Normal Control): UFD 40 mg/kg

Group III (Diabetic Control): Untreated group

Group IV: treated with compound UFD 10 mg/kg

Group V: treated with compound UFD 20 mg/kg

Group VI: treated with compound UFD 40 mg/kg

Group VII: treated with glibenclamide 10 mg/kg.

The treatment continued for 28 days by administration of different doses of UFD and glibenclamide suspended in 0.2% CMC once daily (Nicholas 1956). The fasting blood glucose level was determined day 0, 5, 10, 15, 20, 25 and 28th day. During the experiment period change in the body weight of rat was also recorded.

### Estimation of biochemical parameter

The blood samples were withdrawn on the day 28 collected from a retro orbital puncture technique by capillary tubes containing anticoagulant (disodium ethylene diamine tetra acetate) under mild anesthesia; blood was centrifuged and examined for plasma glucose analysis was done by a GOD - POD method using the Glucose Estimation Kit (Span Diagnostic, India). Other serum estimation was done spectrophotometrically using standard kits which include total cholesterol, HDL and triglyceride (Span Diagnostic, India). Plasma insulin was estimated by the method of reported method of (Nicholas 1956). For determination of the antioxidant enzyme, liver was homogenized in ice chilled 10% potassium chloride solution for estimating different parameters viz. superoxide dismutase (SOD), catalase (CAT), glutathione peroxidase (GPx) and malonaldehyde (MDA) (Sinha 1972; Rotruck et al. 1973; Kakkar et al. 1984).

### Histopathology

For histopathology study, after 28 days all group animals were sacrificed under mild anesthesia and different organs (heart, liver, pancreas and liver) were isolated for histopathological analysis. The isolated organ tissue was fixed at 10% natural buffered formalin, dehydrated by passing through a graded series of alcohol, and embedded in paraffin blocks and 5 mm section was prepared using a semi-automated rotatory microtome. Hematoxylin and eosin were used for staining.

## Results

### Compound identification

The methanolic extract of dried stem bark powder of *A. marmelos* was subjected to column chromatography. Chromatographically identical fractions (having the same R_f_ values) were mixed together and concentrated. Collected fractions were further purified by silica gel recolumn chromatography to isolate compound ‘BG II’ (500 mg). ESI-MS at m/z (rel. int.): 486 [M] + C_21_H_26_O_13_ (2.2), ^1^H NMR (DMSO-d_6_): Table 1; ^13^C NMR (DMSO-d_6_): Table 1; IR _λmax_ (KBr): 3452, 3401, 3325, 2929, 2848, 1702, 1629, 1515, 1457, 1384, 1270, 1118, 1051 cm-1, UV λmax 256, 277, 332 nm (log ϵ 4.1, 5.8, 3.1) (Additional file 1: Spectral Data of umbelliferon-α-D-glucopyranosyl-(2^I^ → 1^II^)-α-D-glucopyranoside).

**Table 1:** ^**13**^ **C NMR spectral data for compounds BG II (UFD)**

Carbon (Position)	δ 1H (***J*** in Hz)	^13^C NMR (DMSO – d_6_)
1	-	-
2	-	162.24
3	6.83	112.51
4	7.47	140.86
5	6.40	122.43
6	7.55	131.20
7	-	158.15
8	7.20	106.37
9	-	116.48
10	-	153.06
1^I^	5.27	103.80
2^I^	4.31	82.31
3^I^	3.80	72.68
4^I^	3.68	69.88
5^I^	4.81	77.89
6^I^	-	61.05
1^II^	4.99	99.61
2^II^	4.02	72.80
3^II^	3.73	71.53
4^II^	3.62	68.91
5^II^	4.48	74.04
6^II^	3.04	60.72

Coupling constants in Hertz are provided in parenthesis.

### Effect of UFD on acute toxicity

Acute toxicity studies exposed the non-toxic nature of the isolated compound UFD. During the acute toxicity study of the UFD on Wistar rats no mortality and no change in the behavior were observed at end of the study. There was no lethality or toxicity found at any selected doses until the end of the study.

### Effect of UFD on oral glucose tolerance test

**Figure 2:**
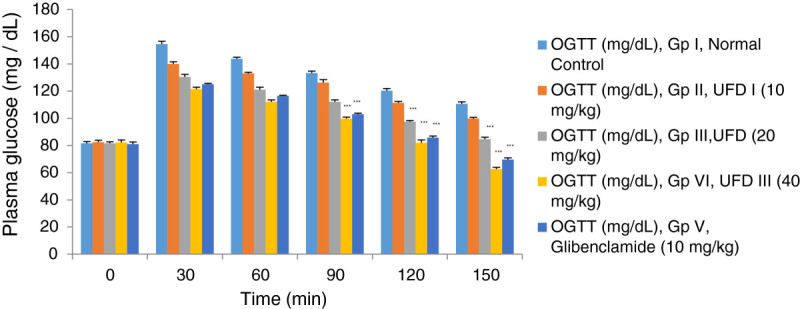
Effect of UFD on fasting plasma glucose on oral glucose tolerance test at different concentrations on STZ induced diabetic rats, compared to standard drug Glibenclamide; values are mean ± SEM; n = 6; *P < 0.05; **P < 0.01; ***P < 0.001; P > 0.05 is considered as non-significant (ns).

The oral glucose tolerance test was evaluated in overnight fasted rats. The effect of different doses of UFD on oral glucose tolerance presented in the Table 2. The starting glucose level of the overnight fasting rat was 81.6 mg/dl. Wistar rats after treated with glucose the levels of blood glucose increased were observed. Treatment was initiated with different doses of UFD and glibenclamide significantly reduced the blood glucose level at 150 min and normalize near to normal control group rat (Figure 2). Significantly, diminishing level of blood glucose was observed with UFD dose 10 mg/kg (28.71%), UFD 20 mg/kg (35.12%), UFD 40 mg/kg (48.43%) and glibenclamide 10 mg/kg (44.32%).

**Table 2:** Effect of UFD on oral glucose tolerance test

S. No.	Groups	Time (min)					
		0	30	60	90	120	150
**1**	**Glucose Control**	81.6 ± 1.208	154.6 ± 1.965	143.8 ± 1.158	133.2 ± 1.463	120.2 ± 1.655	110.6 ± 1.435
**2**	**UFD (10 mg/kg)**	82.4 ± 1.435	140 ± 1.517	133 ± 0.836	126.2 ± 2.289	111.4 ± 0.923	99.8 ± 0.861
**3**	**UFD (20 mg/kg)**	81.6 ± 1.077^**ns**^	130.4 ± 1.991^**ns**^	121 ± 1.817^*^	112 ± 1.517^**^	97.4 ± 0.927^***^	84.6 ± 1.536^***^
**4**	**UFD (40 mg/kg)**	82.2 ± 1.881^**ns**^	121.4 ± 1.503^**^	112 ± 1.517^***^	99.6 ± 1.208^***^	82 ± 2.001^***^	62.6 ± 1.327^***^
**5**	**Glibenclamide (10 mg/kg)**	81 ± 1.581^**ns**^	125 ± 0.707^**^	116.4 ± 0.509^**^	103 ± 0.717^***^	85.8 ± 1.158^***^	69.6 ± 1.248^***^

All values represent mean ± SEM * *P <* 0.05; ** *P <* 0.01; *** *P <* 0.001, ns < non significant; ANOVA, followed by Dunnett’s multiple comparison test.

### Effect of UFD on blood sugar level

**Figure 3:**
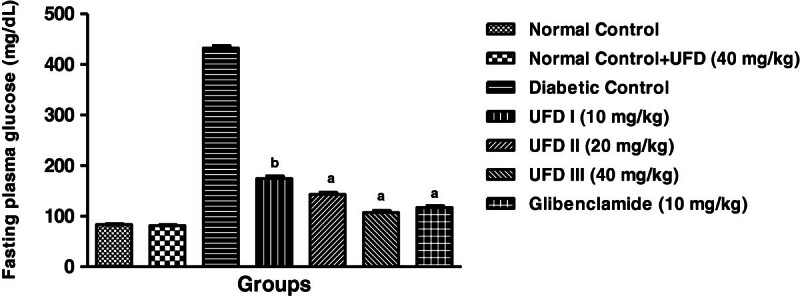
**Effect of UFD on fasting plasma glucose at different concentrations on STZ induced diabetic rats, compared to standard drug Glibenclamide; values are mean ± SEM; n = 6;** ^**c**^ **P < 0.05;** ^**b**^ **P < 0.01;** ^**a**^ **P < 0.001; P > 0.05 is considered as non-significant (ns).**

Administration of STZ produced the diabetes, thereby increase the blood sugar level. The blood glucose level of normal control group rats was 83.4 mg/dl. In the STZ diabetic rats, the level of blood glucose reached to 432.2 mg/dl at day 28. STZ induced diabetic rats treated with different doses of the UFD and glibenclamide showed the significantly lowered the blood glucose level till 28 days. Selected doses of UFD 10, 20 and 40 mg/kg and glibenclamide 10 mg/kg lowered the blood glucose level by 40.84%, 52.33%, 63.45% and 60.45% respectively, thus showing a significant decrease in blood glucose level (Table 3, Figure 3).

**Table 3:** Effect of UFD on biochemical parameter in STZ induced diabetic rats

S. No.	Biochemical parameter	Normal control	Normal control + UFD (40 mg/kg)	STZ-diabetic control^a^	STZ diabetes + UFD (10 mg/kg)^b^	STZ diabetes + UFD (20 mg/kg)^b^	STZ diabetes + UFD (40 mg/kg)^b^	STZ diabetes + Glibenclamide (10 mg/kg)^b^
**1**	Fasting plasma glucose (mg/dL)	83.4 ± 0.509	81.40 ± 0.748	432.4 ± 4.251^***^	174.4 ± 3.945^***^	143 ± 3.082^***^	107.4 ± 2.731^***^	117.2 ± 2.764^***^
**2**	Fasting plasma insulin (μU/mL)	12 ± 0.701	11.8 ± 0.832	2.2 ± 0.374^***^	4.4 ± 0.509^*^	7.2 ± 0.374^**^	10.4 ± 0.519^***^	10.2 ± 0.374^***^
**3**	Glycated heamoglobin (A1c) (%)	1.3 ± 0.071	1.3 ± 0.082	4.52 ± 0.107^***^	3.9 ± 0.172^*^	3.2 ± 0.078^**^	2.34 ± 0.093^***^	2.56 ± 0.075^***^
**4**	Total cholesterol (mg/dl)	65.8 ± 1.281	65.8 ± 0.969	156.6 ± 3.415^***^	101.6 ± 1.375^*^	82 ± 0.7071^**^	68.4 ± 1.691^***^	72.4 ± 1.364^***^
**5**	Triglycerides (mg/dl)	83 ± 1.732	83.6 ± 1.412	156.8 ± 4.247^***^	116.4 ± 1.721^*^	109.2 ± 1.801^**^	91.6 ± 2.315^***^	95.8 ± 2.354^***^
**6**	HDL cholesterol (mg/dl)	54.8 ± 2.417	55.4 ± 1.536	26.6 ± 1.364^***^	37 ± 1.304^*^	42.8 ± 1.715^**^	53.2 ± 1.463^***^	50.6 ± 1.077^***^
**7**	LDL cholesterol (mg/dl)	11.40 ± 0.245	10 ± 0.316	151.6 ± 0.509^***^	74.8 ± 0.374^*^	49 ± 0.316^**^	21.4 ± 1.913^***^	27 ± 0.316^***^
**8**	VLDL cholesterol (mg/dl)	16.6 ± 0.346	16.72 ± 0.281	31.36 ± 0.849^***^	23.28 ± 0.344^*^	21.84 ± 0.361^**^	18.32 ± 0.463^***^	19.16 ± 0.471^***^
**9**	Hexokinase (μg/mg of tissue)	147.2 ± 2.498	147 ± 2.302	96 ± 2.429^***^	112 ± 1.141^*^	128.6 ± 3.251^**^	141.4 ± 1.913^***^	137.4 ± 1.327^***^
**10**	Glucose-6-phosphatase (unit/mg of tissue)	9.2 ± 0.583	9.2 ± 0.583	14.2 ± .582^***^	13.6 ± 0.401^**ns**^	11.4 ± 0.509^*^	9.8 ± 0.374^***^	10.8 ± .372^***^
**11**	Fructose-1-6-biphosphatase (unit/mg of tissue)	29.6 ± 0.927	29.80 ± 0.969	55.6 ± 1.077^***^	45.4 ± 0.927^*^	35 ± 0.707^**^	25.6 ± 0.509^***^	29 ± 0.707^***^
**12**	Weight variation (g)	202.2 ± 1.021	206.2 ± 2.035	155.6 ± 3.011^***^	193.8 ± 2.267^***^	199.4 ± 1.435^***^	203.4 ± 1.778^***^	200.4 ± 1.722^***^

All values represent mean ± SEM * *P <* 0.05; ** *P <* 0.01; *** *P <* 0.001, ns < non significant; ANOVA, followed by Dunnett’s multiple comparison test.

^a^ Compared to vehicle control.

^b^ Compared to diabetic control.

### Effect of UFD on body weight

**Figure 4:**
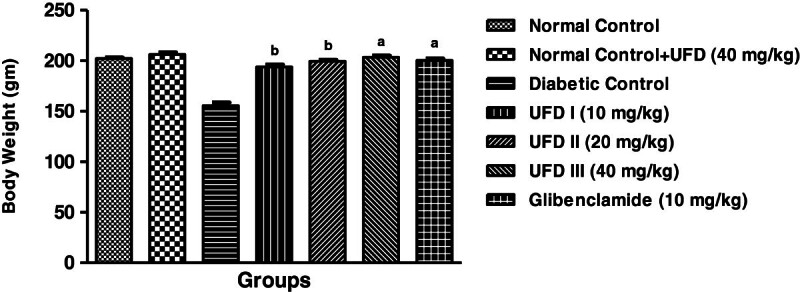
**Effect of UFD on body weight at different concentrations on STZ induced diabetic rats, compared to standard drug Glibenclamide; values are mean ± SEM; n = 6;** ^**c**^ **P < 0.05;** ^**b**^ **P < 0.01;** ^**a**^ **P < 0.001; P > 0.05 is considered as non-significant (ns).**

The initial body weight was similar in non diabetic as well as diabetic control groups. The administration of different doses of UFD and glibenclamide treated group significantly (P < 0.001) prevented decrease in body weight (Table 3, Figure 4).

### Effect of UFD on plasma insulin level

**Figure 5:**
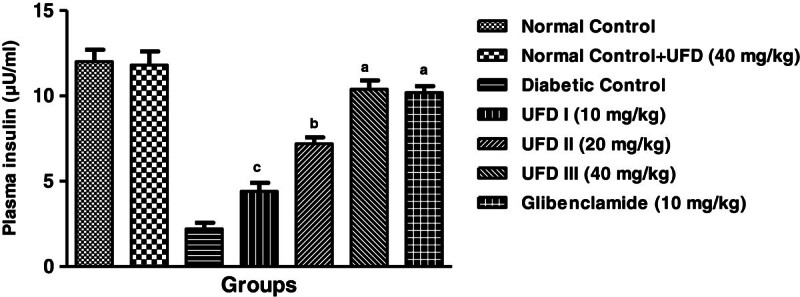
**Effect of UFD on level of plasma insulin at different concentrations on STZ induced diabetic rats, compared to standard drug Glibenclamide; values are mean ± SEM; n = 6;** ^**c**^ **P < 0.05;** ^**b**^ **P < 0.01;** ^**a**^ **P < 0.001; P > 0.05 is considered as non-significant (ns).**

The serum insulin level significantly decreased in STZ induced diabetic control group rats was observed. Three different doses of UFD (10, 20 and 40 mg/kg) and glibenclamide (10 mg/kg) treated group rats showed significant (P < 0.001) increase in the pancreatic insulin compared to diabetic control group rats on day 28 (Table 3, Figure 5).

### Effect of UFD on the level of glycated hemoglobin

**Figure 6:**
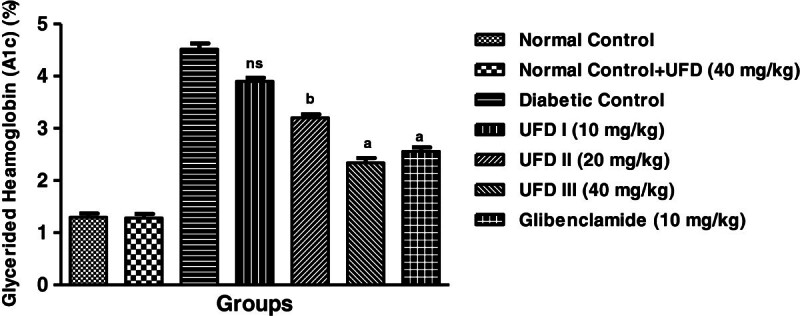
**Effect of UFD on level of glycated hemoglobin (A1c) (%) at different concentrations on STZ induced diabetic rats, compared to standard drug Glibenclamide; values are mean ± SEM; n = 6;** ^**c**^ **P < 0.05;** ^**b**^ **P < 0.01;** ^**a**^ **P < 0.001; P > 0.05 is considered as non-significant (ns).**

The level of glycated hemoglobin was increased in diabetic group rats. Three different doses of UFD (10, 20 and 40 mg/kg) and glibenclamide (10 mg/kg) treated group rats, significantly (P < 0.001) decrease in the level of glycated hemoglobin compared to diabetic control groups rats on day 28 was observed (Table 3, Figure 6).

### Effect of UFD on the level of hexokinase

**Figure 7:**
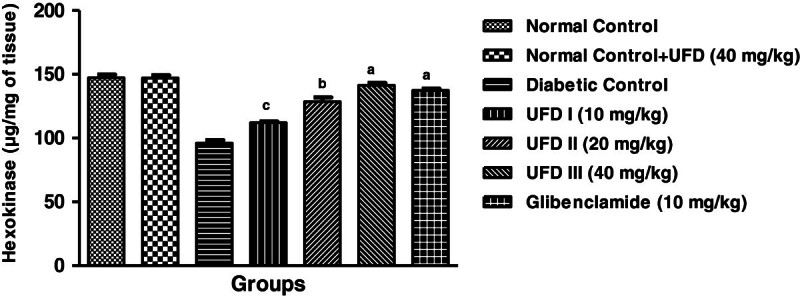
**Effect of UFD on level of Hexokinase at different concentrations on STZ induced diabetic rats, compared to standard drug Glibenclamide; values are mean ± SEM; n = 6;** ^**c**^ **P < 0.05;** ^**b**^ **P < 0.01;** ^**a**^ **P < 0.001; P > 0.05 is considered as non-significant (ns).**

The level of the hexokinase was decreased in STZ induced diabetic rats. Three different doses of UFD (10, 20 and 40 mg/kg) and glibenclamide (10 mg/kg) treated group rats, significantly (P < 0.001) increasing the level of hexokinase compared to diabetic control groups rats on day 28 (Table 3, Figure 7). The level of hexokinase at UFD doses 40 mg/kg was a maximum intensification at compared to other group received different doses of UFD and glibenclamide.

### Effect of UFD on the levels of glucose-6-phosphatase

**Figure 8:**
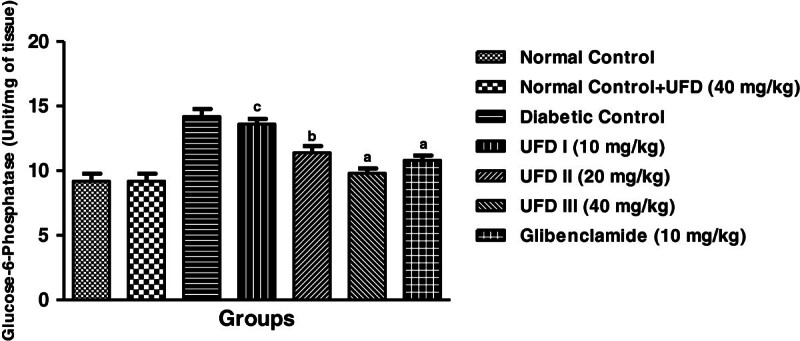
**Effect of UFD on level of Glucose-6-phosphatase at different concentrations on STZ induced diabetic rats, compared to standard drug Glibenclamide; values are mean ± SEM; n = 6;** ^**c**^ **P < 0.05;** ^**b**^ **P < 0.01;** ^**a**^ **P < 0.001; P > 0.05 is considered as non-significant (ns).**

In the STZ induced diabetic rats increased the level of glucose-6-phosphate. Three different doses of UFD (10, 20 and 40 mg/kg) and glibenclamide (10 mg/kg) treated groups rats, significantly (P < 0.001) decreased the level of glucose-6-phosphate compared to diabetic control groups rats on day 28 (Table 3, Figure 8). An UFD dose 40 mg/kg was more effective dose as compared to the different doses of UFD and glibenclamide administration groups.

### Effect of UFD on the levels of fructose-1-6-biphosphatase

**Figure 9:**
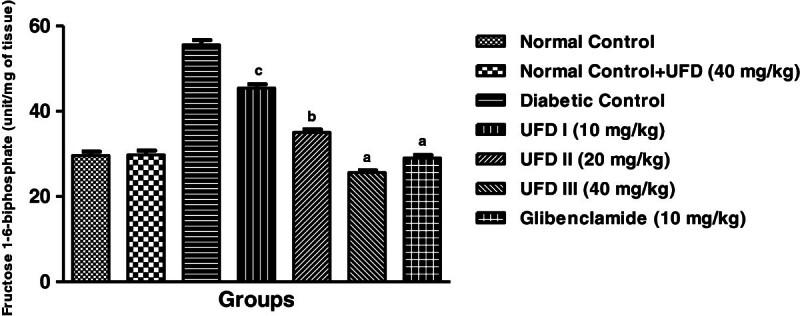
**Effect of UFD on level of Fructose1-6-biphosphate at different concentrations on STZ induced diabetic rats, compared to standard drug Glibenclamide; values are mean ± SEM; n = 6;** ^**c**^ **P < 0.05;** ^**b**^ **P < 0.01;** ^**a**^ **P < 0.001; P > 0.05 is considered as non-significant (ns).**

To evaluate the effect of different doses of UFD on distressed hepatic activity, we administered UFD to STZ induced diabetic rats. The level of fructose-1-6-biphosphate was reached higher in diabetic rats. The administration of different doses of UFD (10, 20 and 40 mg/kg) and glibenclamide (10 mg/kg) treated groups rats significantly (P < 0.001) declining the level of fructose-1-6-biphosphatse (Table 3, Figure 9).

### Effect of UFD on the level of total cholesterol

**Figure 10:**
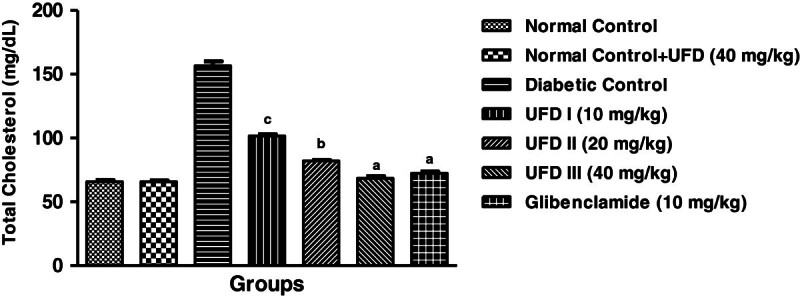
**Effect of UFD on level of total cholesterol at different concentrations on STZ induced diabetic rats, compared to standard drug Glibenclamide; values are mean ± SEM; n = 6;** ^**c**^ **P < 0.05;** ^**b**^ **P < 0.01;** ^**a**^ **P < 0.001; P > 0.05 is considered as non-significant (ns).**

The level of cholesterol was increased in the STZ induced diabetic rats. The administration of different doses of UFD significantly decreased the level of total cholesterol (Table 3, Figure 10).

### Effect of UFD on the levels of serum triglycerides

**Figure 11:**
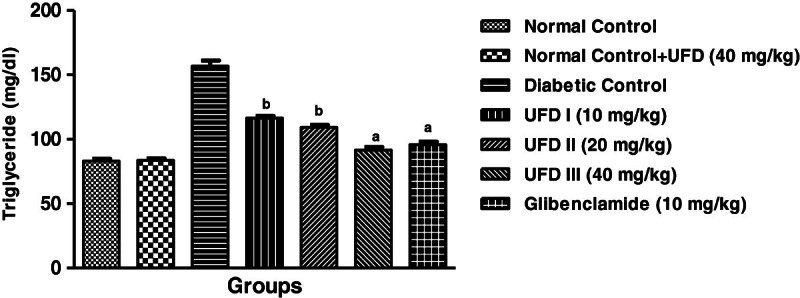
**Effect of UFD on level of triglyceride at different concentrations on STZ induced diabetic rats, compared to standard drug Glibenclamide; values are mean ± SEM; n = 6;** ^**c**^ **P < 0.05;** ^**b**^ **P < 0.01;** ^**a**^ **P < 0.001; P > 0.05 is considered as non-significant (ns).**

It is evident from the figure that the administrations of STZ to Wistar (albino strain) rats show an increase in the serum triglyceride level. The administration of different doses of UFD the level of serum triglyceride subordinate to a good extent. The maximum lowering the serum triglyceride was appeared in the group received UFD at a dose (40 mg/kg) (Table 3, Figure 11).

### Effect of UFD on the level of HDL cholesterol

**Figure 12:**
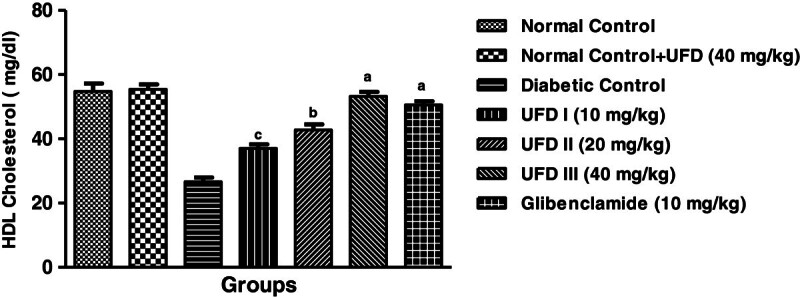
**Effect of UFD on level of HDL (High density lipoprotein) cholesterol at different concentrations on STZ induced diabetic rats, compared to standard drug Glibenclamide; values are mean ± SEM; n = 6;** ^**c**^ **P < 0.05;** ^**b**^ **P < 0.01;** ^**a**^ **P < 0.001; P > 0.05 is considered as non-significant (ns).**

It is predictable that the level of HDL cholesterol was decreased in the STZ diabetic rats. Upon the administration of different doses of UFD, significant increase in the level of HDL cholesterol as compared to the diabetic rats (Table 3, Figure 12).

### Effect of UFD on the level of LDL cholesterol

**Figure 13:**
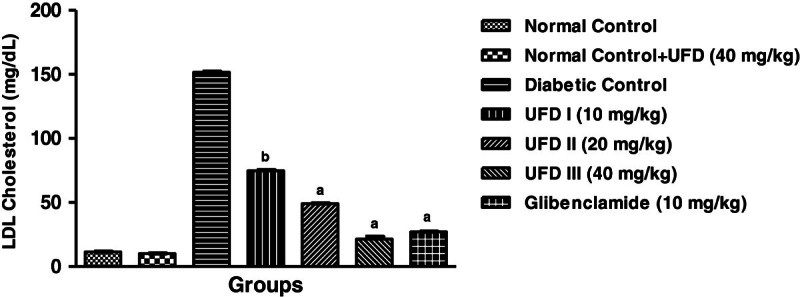
**Effect of UFD on level of LDL (Low density lipoprotein) cholesterol at different concentrations on STZ induced diabetic rats, compared to standard drug Glibenclamide; values are mean ± SEM; n = 6;** ^**c**^ **P < 0.05;** ^**b**^ **P < 0.01;** ^**a**^ **P < 0.001; P > 0.05 is considered as non-significant (ns).**

It is evident from the Figure 13 that STZ induced diabetic rat showed an increase in level of LDL cholesterol. Treatment with different doses of UFD decreases the level of LDL cholesterol. The figure suggests that maximum decrease in the higher level of LDL cholesterol was found in UFD (40 mg/kg) dose (Table 3).

### Effect of UFD on the level of VLDL cholesterol

**Figure 14:**
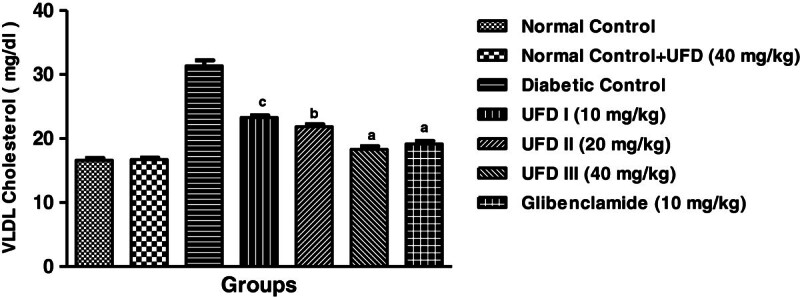
**Effect of UFD on level of VLDL (Very low density lipoprotein) cholesterol at different concentrations on STZ induced diabetic rats, compared to standard drug Glibenclamide; values are mean ± SEM; n = 6;** ^**c**^ **P < 0.05;** ^**b**^ **P < 0.01;** ^**a**^ **P < 0.001; P > 0.05 is considered as non-significant (ns).**

STZ induced diabetic rat clearly depicted the increased level of VLDL cholesterol (Table 3). Oral administration of different doses of UFD and glibenclamide significantly (P < 0.001) decreases the level of VLDL cholesterol. The Figure 14 suggests that UFD (40 mg/kg) dose was more effective in decreasing the elevated level of VLDL cholesterol.

### Effect of UFD on enzymatic antioxidant markers

**Figure 15:**
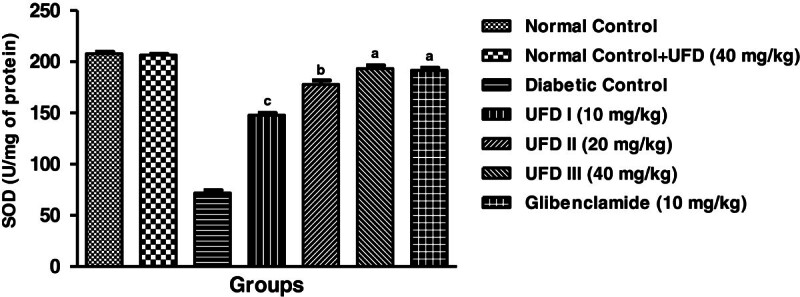
**Effect of UFD on level of SOD (Superoxide dismutase) cholesterol at different concentrations on STZ induced diabetic rats, compared to standard drug Glibenclamide; values are mean ± SEM; n = 6;** ^**c**^ **P < 0.05;** ^**b**^ **P < 0.01;** ^**a**^ **P < 0.001; P > 0.05 is considered as non-significant (ns).**

**Figure 16:**
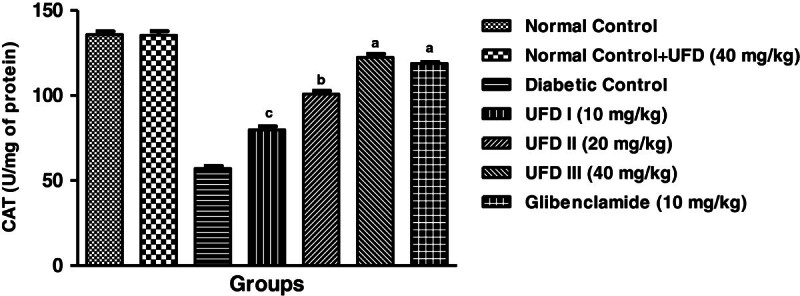
**Effect of UFD on level of CAT (Catalase) cholesterol at different concentrations on STZ induced diabetic rats, compared to standard drug Glibenclamide; values are mean ± SEM; n = 6;** ^**c**^ **P < 0.05;** ^**b**^ **P < 0.01;** ^**a**^ **P < 0.001; P > 0.05 is considered as non-significant (ns).**

**Figure 17:**
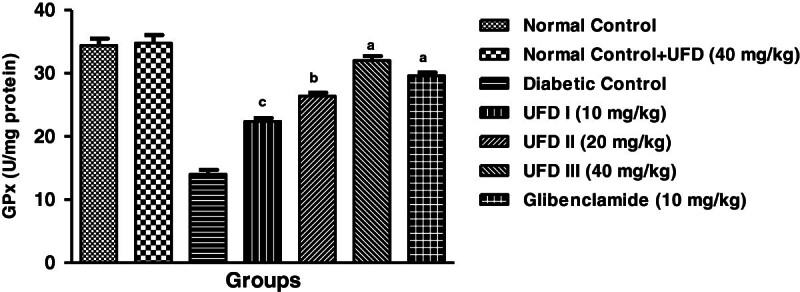
**Effect of UFD on level of GPx (Glutathione peroxidase) cholesterol at different concentrations on STZ induced diabetic rats, compared to standard drug Glibenclamide; values are mean ± SEM; n = 6;** ^**c**^ **P < 0.05;** ^**b**^ **P < 0.01;** ^**a**^ **P < 0.001; P > 0.05 is considered as non-significant (ns).**

**Figure 18:**
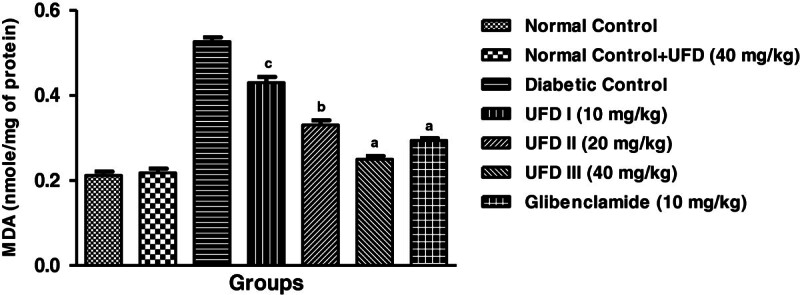
**Effect of UFD on level of MDA (Malondialdehyde) cholesterol at different concentrations on STZ induced diabetic rats, compared to standard drug Glibenclamide; values are mean ± SEM; n = 6;** ^**c**^ **P < 0.05;** ^**b**^ **P < 0.01;** ^**a**^ **P < 0.001; P > 0.05 is considered as non-significant (ns).**

Affect on enzymatic antioxidant markers, the level of superoxidase dismutase (SOD), glutathione peroxidase (GPx) and catalase (CAT) were increased and the level of malondialdehyde (MDA) was significantly decreased in STZ induced diabetic groups rat. Treatment with the different doses of UFD (10, 20 and 40 mg/kg) and glibenclamide (10 mg/kg) treated group rats significantly (P < 0.001) increased the level of SOD (Figure 15), GPx (Figure 16), CAT (Figure 17) and decreased the level of MDA (Table 4, Figure 18).

**Table 4:** Effect UFD on antioxidant enzyme at end of the study

S. No.	Biochemical parameter	Normal control	Normal control + UFG (40 mg/kg)	STZ-diabetic control^a^	STZ diabetes + UFD (10 mg/kg)^b^	STZ diabetes + UFD (20 mg/kg)^b^	STZ diabetes + UFD (40 mg/kg)^b^	STZ diabetes + Glibenclamide (10 mg/kg)^b^
**1**	SOD (U/mg of protein)	207.8 ± 1.985	206.6 ± 1.077	71.8 ± 2.691^***^	147.8 ± 2.177^*^	177.8 ± 3.955^**^	193.2 ± 3.247^***^	191.6 ± 2.421^***^
**2**	CAT (U/mg of protein)	135.8 ± 1.855	135.4 ± 2.358	57 ± 1.517^***^	79.8 ± 1.985^*^	100.8 ± 1.934^*^	122.4 ± 2.015^***^	118.8 ± 0.861^***^
**3**	GPx (nmole/mg of protein)	34.4 ± 1.077	34.8 ± 1.241	14 ± 0.707^***^	22.4 ± 0.509^*^	26.4 ± 0.562^**^	32 ± 0.712^***^	29.6 ± 0.514^***^
**4**	MDA (nmole/mg of protein)	0.212 ± 0.008	0.218 ± 0.009	0.526 ± 0.011^***^	0.431 ± 0.013^*^	0.331 ± 0.012^**^	0.251 ± 0.007^***^	0.294 ± 0.005^***^

All values represent mean ± SEM * *P <* 0.05; ** *P <* 0.01; *** *P <* 0.001, ns < non significant; ANOVA, followed by Dunnett’s multiple comparison test.

^a^Compared to vehicle control.

^b^Compared to diabetic control.

### Effect of UFD on liver histopathology

**Figure 19:**
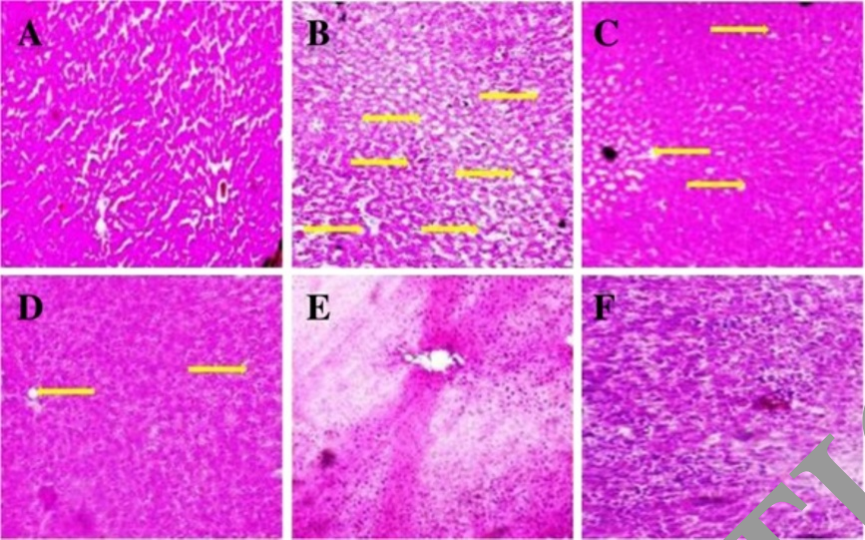
**Effect of UFD on liver in different groups of rats:** **(A) Normal control: Histopathology of normal control group did not shown any fat deposition and other changes (B) Diabetic control: Histopathology of diabetic rat showed accumulation of micro droplet of fat (yellow arrow) (C)** **UFD I (10 mg/kg): Histopathology of the starting dose of tested drug shown some part having fat deposition which was less compared to the diabetic control (yellow arrow).** **(D)** UFD II (20 mg/kg): Histopathology of second dose of tested drug showed very few micro droplet of fat (yellow arrow). **(E)** UFD III (40 mg/kg): Histopathology of other tested drug not showing any fat deposition and other changes. **(F)** Glibenclamide (10 mg/kg): Standard drug treated group shown histopathology similar to the normal control groups. The samples were obtained from the same liver anatomical regions. For each group, 6 rats were examined and 80 pictures were taken. The above picture for each group was chosen randomly from the 80 pictures in this group. Original magnification, 10 × .

**Figure 20:**
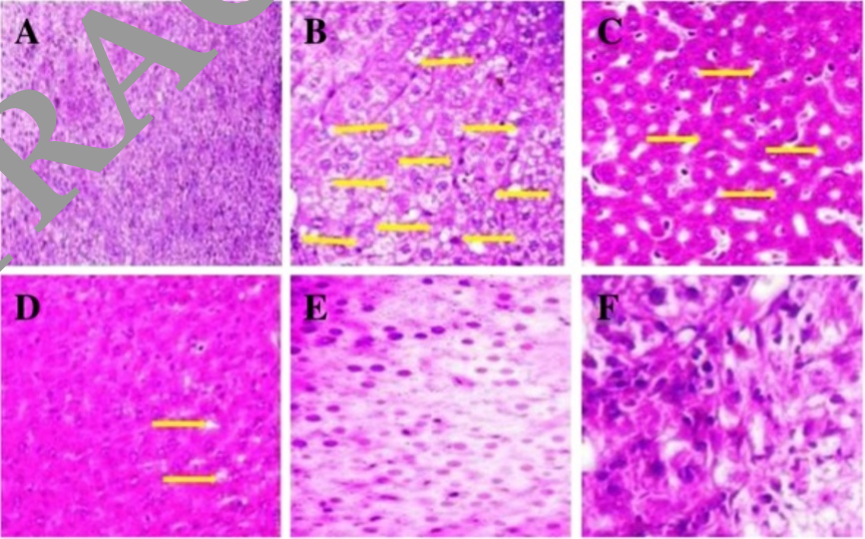
**Effect of UFD on liver in different groups of rats: (A) normal control: Normal control not showing any change in liver histopathology. (B)** Diabetic control: Diabetic control rat shown fat deposition on the liver in the form of micro droplet. (yellow arrow) **(C)** UFD I (10 mg/kg): Tested drug some part showed the deposition of micro droplet of fat (yellow arrow). **(D)** UFD II (20 mg/kg): Histopathology of tested drug showed some part of micro droplet of fat deposition (yellow arrow). **(E)** UFD III (40 mg/kg): Histopathology of tested drug similar to the glibenclamide treated drug. **(F)** Glibenclamide (10 mg/kg): Standard drug treated group shown histopathology similar to the normal control groups. The samples were obtained from the same liver anatomical regions. For each group, 6 rats were examined and 80 pictures were taken. The above picture for each group was chosen randomly from the 80 pictures in this group. Original magnification, 40 × .

Histopathology studies of the STZ induced diabetic rat showed increased level of fat accumulation and large area of hepatocytes taken over by fat droplet (Figure 19). Oral administration of UFD with different doses improved the kidney histopathology. UFD dose (10 mg/kg) showed deposition of fat as compared to the normal rat, UFD dose 20 mg/kg histopathology showed few macro droplets of fat and UFD dose 40 mg/kg shown no fat deposition as shown in the liver histopathology. Glibenclamide treated group rat histopathology had shown normal liver (Figure 20).

### Effect of UFD on kidney histopathology

**Figure 21:**
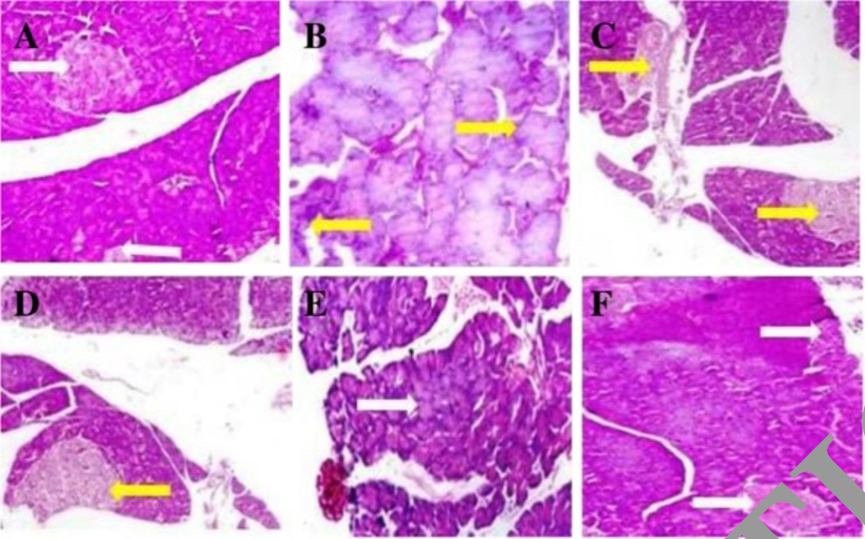
**Effect of UFD photomicrographs of histological changes in rat pancreas: (A) Normal control: normal histological structure of rat pancreas showed normal islet (white arrow) (B) Diabetic control: Histopathology of diabetic control rat showed focal necrosis (yellow arrow) (C) UFD I (10 mg/kg): Histopathology of tested drug rat showed bigger size of islet and focal necrosis (yellow arrow) (D) UFD II (20 mg/kg): Histopathology of tested drug rat showed focal necrosis (yellow arrow) (E) UFD III (40 mg/kg): Histopathology of tested drug rat showed normal size of islet (white arrow) (F) Glibenclamide (10 mg/kg): glibenclamide treated rat pancreas showed normal islet (white arrow).** For each group 6 rats were examined and 80 pictures were taken. The above picture for each group was chosen randomly from the 80 pictures in this group. Original magnification, 10 × .

**Figure 22:**
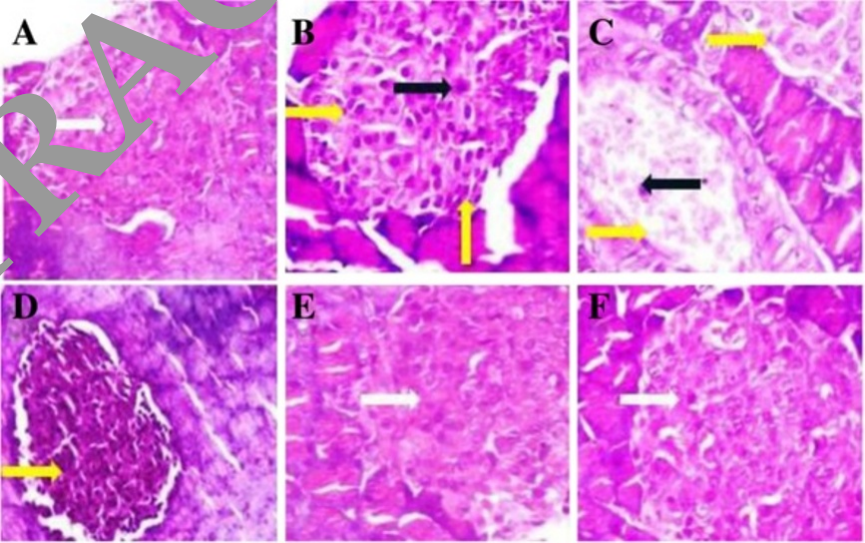
**Effect of UFD photomicrographs of histological changes in rat pancreas: (A) Normal control: normal histological structure of rat pancreas showed normal islet (white arrow) (B) Diabetic control: Histopathology of diabetic control rat showed focal necrosis (yellow arrow) (C) UFD I (10 mg/kg): Histopathology of tested drug rat showed bigger size of islet and focal necrosis (yellow arrow) (D) UFD II (20 mg/kg): Histopathology of tested drug rat showed focal necrosis (yellow arrow) (E) UFD III (40 mg/kg): Histopathology of tested drug rat showed normal size of islet (white arrow) (F) Glibenclamide (10 mg/kg): glibenclamide treated rat pancreas showed normal islet (white arrow).** For each group 6 rats were examined and 80 pictures were taken. The above picture for each group was chosen randomly from the 80 pictures in this group. Original magnification, 40 × .

Histopathology of STZ induced diabetic rat kidney shows inflammation in blood vessels, fat deposition, changes in size of glomerulus and increases the thickness of bowman capsules. Oral treatment with different doses of UFD and glibenclamide showed the changes in STZ induced diabetic groups rat. Different doses of UFD showing less fat deposition, normal size of glomerulus and bowman capsules at dose dependent manner. The UFD (40 mg/kg) dose showed normal histopathology of the kidney as compared to the normal control (Figures 21 and 22).

### Effect of UFD on pancreas histopathology

**Figure 23:**
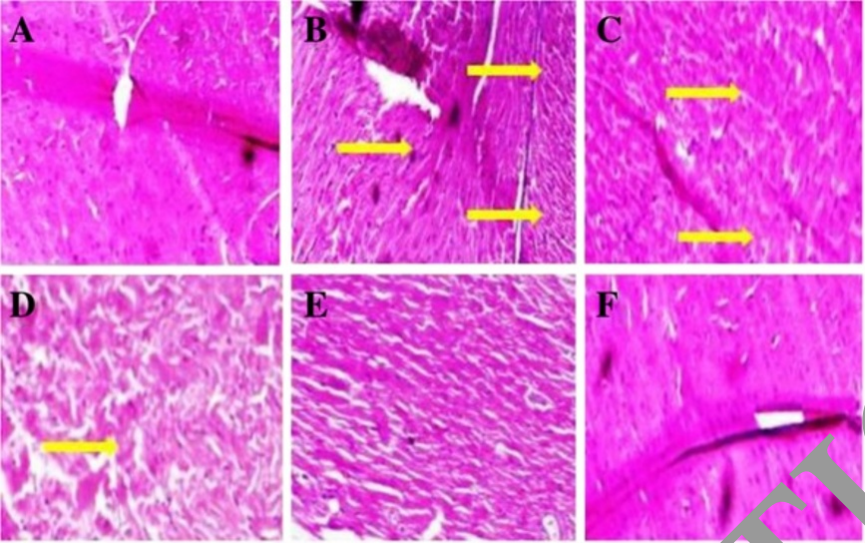
**Effect of UFD photomicrographs of histological on heart in different groups of rats: (A) Normal control: Histopathology of normal control group rat normal histopathology of heart (B) Diabetic control: Histopathology of diabetic control group rat showed increased interstitial space and distort the intercalated disc (yellow arrow) (C) UFD I (10 mg/kg): Histopathology of tested drug shown decreased interstitial space and intercalated disc (yellow arrow) (D) UFD II (20 mg/kg): Histopathology of tested drug showed less interstitial space (yellow arrow) (E) UFD III (40 mg/kg): Histopathology of tested drug showed normal heart like the glibenclamide (F) Glibenclamide (10 mg/kg): Histopathology of glibenclamide treated drug shown the normal histopathology of heart.** The samples were obtained from the same liver anatomical regions. For each group, 6 rats were examined and 80 pictures were taken. The above picture for each group was chosen randomly from the 80 pictures in this group. Original magnification, 10 × .

**Figure 24:**
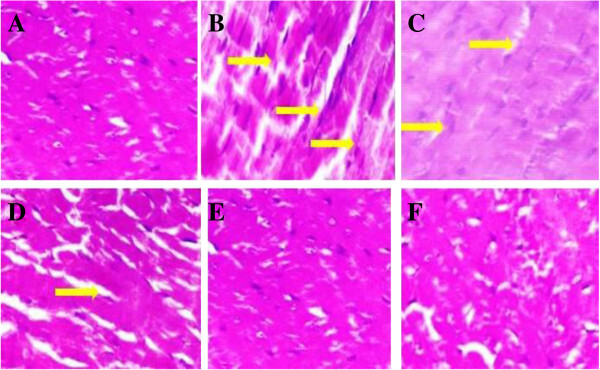
**Effect of UFD photomicrographs of histological on heart in different groups of rats: (A) Normal control: Histopathology of normal control group rat normal histopathology of heart (B) Diabetic control: Histopathology of diabetic control group rat showed increased interstitial space and distort the intercalated disc (yellow arrow) (C) UFD I (10 mg/kg): Histopathology of tested drug showed decreased interstitial space and intercalated disc (yellow arrow) (D) UFD II (20 mg/kg): Histopathology of tested drug showed less interstitial space (yellow arrow) (E) UFD III (40 mg/kg): Histopathology of tested drug shown normal heart like the glibenclamide (F) Glibenclamide (10 mg/kg): Histopathology of glibenclamide treated drug showed the normal histopathology of heart.** The samples were obtained from the same liver anatomical regions. For each group, 6 rats were examined and 80 pictures were taken. The above picture for each group was chosen randomly from the 80 pictures in this group. Original magnification, 40 × .

Histopathology studies of pancreas of STZ induced diabetic rat displayed reduction of the Islets of Langerhans, damaged or reduced the size of β cells and extensive necrosis changes followed by fibrosis and atrophy. STZ induced diabetic rat treated with different doses of UFD and glibenclamide restored the necrotic and fibrotic changes and raised the number of β cells (Figures 23 and 24).

### Effect of UFD on heart histopathology

**Figure 25:**
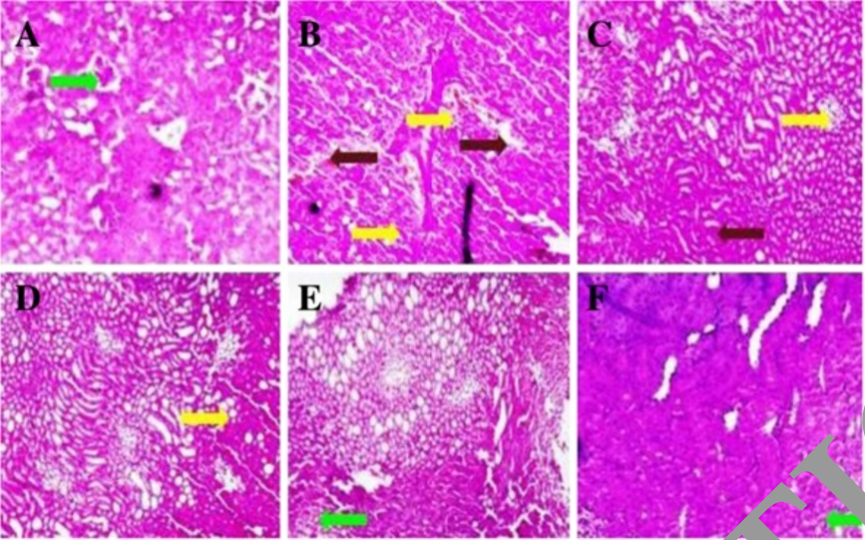
**Effect of UFD on kidney in different groups of rat: (A) Normal control: Normal control histopathology showed normal size of glomerulus (green arrow) (B) Diabetic control: Histopathology of diabetic control rat showed inflammatory cell in blood vessels (brown arrow) and deposition of fats (yellow arrow) (C) UFD I (10 mg/kg): Histopathology of tested drug showed inflammation in blood vessels (brown arrow) and fat deposition (yellow arrow) (D) UFD II (20 mg/kg): Histopathology of tested drug showed only fat deposition (yellow arrow) (E) UFD III (40 mg/kg): Histopathology of tested drug showed normal kidney histopathology like the glibenclamide group (F) Glibenclamide (10 mg/kg): Glibenclamide treated animal histopathology shown the normal kidney.** The samples were obtained from the same liver anatomical regions. For each group, 6 rats were examined and 80 pictures were taken. The above picture for each group was chosen randomly from the 80 pictures in this group. Original magnification, 10 × .

**Figure 26:**
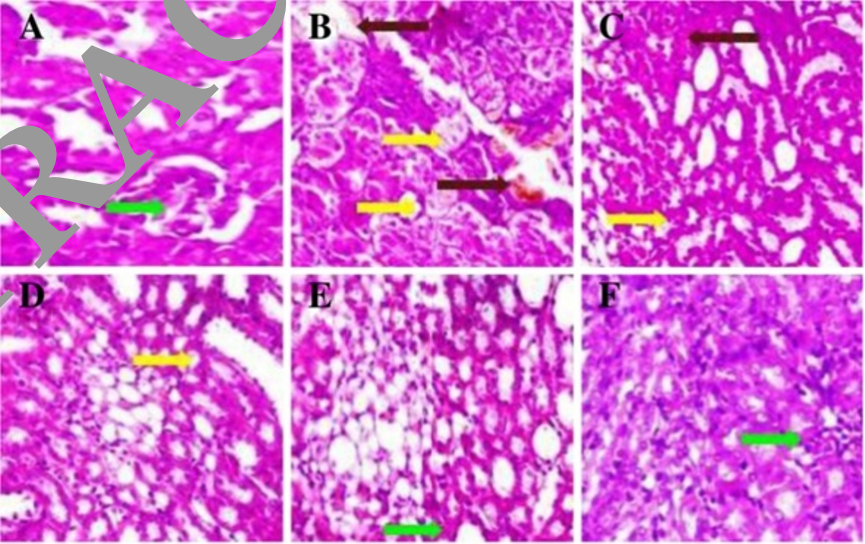
**Effect of UFD on kidney in different groups of rat: (A) Normal control: Normal control histopathology showed average size of glomerulus (green arrow) (B) Diabetic control: Diabetic control histopathology showed fat deposition (yellow arrow) inflammatory cell in blood vessels (brown arrow) (C) UFD I (10 mg/kg): Histopathology of tested drug showed inflammation in blood vessels (brown arrow) and fat deposition (yellow arrow) (D) UFD II (20 mg/kg): Histopathology of tested drug showed fat deposition in few place (yellow arrow) (E) UFD III (40 mg/kg): Histopathology of tested drug showed normal glomerulus but slightly bigger in size (F) Glibenclamide (10 mg/kg): Glibenclamide treated animal histopathology shown the normal kidney.** The samples were obtained from the same liver anatomical regions. For each group, 6 rats were examined and 80 pictures were taken. The above picture for each group was chosen randomly from the 80 pictures in this group. Original magnification, 40 × .

STZ induced diabetic rat showed the increased degree of interstitial space and distort intercalated disc (Figure 25). STZ induced diabetic rat, treatment with altered doses of UFD and glibenclamide showed effect on the heart histopathology. STZ induced diabetic rat treated with UFD (10 mg/kg) dose showed less interstitial space and distort intercalated disc compared to the diabetic control, other UFD (20 mg/kg) dose showing some interstitial space and UFD (40 mg/kg) dose showed the normal histopathology of heart like glibenclamide treated group rat (Figure 26). Glibenclamide treated group exhibited the histopathology like the normal control.

## Discussion

*Aegle marmelos* Correa rich source of many compounds. The methanolic extract was subjected to column chromatography and isolated the compound. The isolated compound exhibited blue fluorescence and UV absorption maxima at 256, 277 and 332 nm and IR absorption band at 1702 cm^-1^ for δ-lactone ring suggested coumarin nature of the molecule. It also had IR absorption bands for hydroxyl groups (3452, 3401, 3325 cm^-1^) and an aromatic ring (1629, 1515 cm^-1^). On the basis of mass spectrum and ^13^C NMR spectra the molecular ion peak of the compound was determined at m/z 486 consistent to the molecular formula of a coumarin diglycoside C_21_H_26_O_13_. The ^1^H NMR spectrum showed the presence of two AB-type doublets at δ 6.83 (J = 9.2 Hz) and 7.47 (J = 9.2 Hz) assigned to vinylic H-3 and H-4 protons, respectively. A one-proton double doublet at δ 7.55 (J = 9.8, 2.8 Hz) and two one-proton doublets at δ 7.20 (J = 2.8 Hz) and 6.40 Hz (J = 9.8 Hz) were ascribed to coumarin H-6, H-8 and H-5 protons, respectively. Two one-proton doublets at δ 5.27 (J = 3.6 Hz) and 4.99 (J = 3.6 Hz) were accounted to α-oriented anomeric H-1^I^ and H-1^II^ protons, respectively. The other sugar protons resonated between δ 4.81 – 3.04. The ^13^C NMR spectrum displayed signals for nine coumarin carbons in the range of δ 162.24 – 106.36, anomeric carbons at δ 103.80 (C-1^I^), 99.61 (C-1^II^) and other sugar carbons between δ 82.31 – 60.72. The existence of NMR H-2^I^ signal in the deshielded region at δ 4.31 and carbon C-2^I^ signal at δ 82.31 indicated (2^I^ → 1^II^) linkage of the sugar units. The HMBC spectrum of the coumarin showed interactions of H-6, H-8 and H-1I with C-7; H-3 and H-4 with C-2; and H-2^I^, H-2^II^ and H-3^II^ with C-1^II^. The ^1^H and ^13^C NMR spectral data of the coumarin nucleus were compared with the reported data of other coumarins (Rao et al. 2009; Aslam et al. 2012; Chakthong et al 2012). On the basis of spectral data analysis the structure of this compound has been elucidated as umbelliferon-α-D-glucopyranosyl-(2^I^ → 1^II^)-α-D-glucopyranoside.

Diabetes (Type II) generally occurs due to human genetically susceptibility, as a result loss of insulin producing pancreatic β-cell cytotoxicity mediated through the release of nitric oxide (NO). Insulin dependent diabetes mellitus (IDDM) is caused by the progressive destruction of the insulin secreting pancreatic β-cells. STZ is a cytotoxic compound obtained from the soil microbes *Streptomyces achromogenes*. STZ mainly penetrate the β-cells via glucose transporter and break the DNA strand in β-cells causing the endogenous insulin release (Kumar et al. 2011b). Due to breakage of DNA strand leads to amendment of blood sugar level and glucose concentrations in blood. Several plant have been accounted as an antidiabetic effect by a variety of mechanisms such as stimulating the regeneration of Islets of Langerhans in the pancreas, improving insulin sensitivity and augmenting glucose dependent insulin secretion in STZ induced diabetic rats (Sezik et al. 2005; Daisy et al. 2009).

A lot of synthetic antidiabetic drugs available in the market but sulfonylurea such as glibenclamide often use as a standard antidiabetic drug in STZ induced diabetes to compare the efficacy of a variety of antihyperglycemic compounds. (Kumar et al. 2013a).

Acute toxicity studies of the bioactive compound of UFD revealed the non-toxic nature in the lower dose. There was no lethality or any toxic reactions found with the selected doses of UFD until the end of the specific study. The selection of the doses was done on the basis of calibration curve (Salahuddin and Jalalpure 2010).

Oral glucose tolerance test was performed for the identification of the alteration of carbohydrate metabolism during post glucose administration. The different doses of the UFD significantly altered the blood glucose level as compared to the glucose control group rats. The result suggests that the different doses of the UFD have better glucose utilization capacity. The possible mechanism of action of the UFD may be due to insulin emission from the β-cell and improved the glucose transportation and consumption in the rats (Ceriello 2005: Santiagu et al. 2012).

STZ induced diabetic rat showed the increase level of the blood glucose and decrease level of the plasma insulin. STZ destroy the β-cell in the pancreas and increase the overproduction of glucose and gluconeogenesis. Gluconeogenesis and overproduction of the glucose is the prime factor of the hyperglycemia (Latner 1958). STZ induced diabetic rats treated with the different doses of the UFD significantly decreased the blood glucose level and improve the plasma insulin level by regeneration of the β-cells. The possible mechanism of action of the UFD may be stimulating the insulin secretion and regeneration of the β-cells of the pancreas or regeneration of the granules in the β-cells and enhanced the cellularity of the Islet of Langerhans (Kumar et al. 2013b). The hypothesis further confirmed by the pancreas histopathology which showed that the UFD exhibit the protective effect over the pancreas against the microbial streptozotocin (Figures 23 and 24). The UFD shows the similar mechanism of action as glibenclamide, stimulating the insulin secretion.

The decrease in body weight was found throughout the study in diabetic control group rats. The decrease in body weight due to gluconeogenesis, catabolism of proteins and fats. Catabolism which is directly associated with the characteristic loss of body weight due to increased muscle destruction or degradation of structural proteins (Paulsen 1973; Shirwaikar et al. 2004; Shirwaikar et al. 2006). In this manuscript, results suggest that STZ induced diabetic groups rats treated with different doses of UFD significantly increased the body weight as compared to the diabetic control group rats in dose dependent manner. The potential mechanism of action of the UFD showed the protective effect against the controlling the muscle wasting (reversal of gluconeogenesis).

STZ induced diabetic rats showed the blood glucose level increased, increase level of glucose, glucose add to the RBC in N terminal of hemoglobin chain and producing the glycated hemoglobin (Hba1c) and increased the level of glycated hemoglobin in STZ induced diabetic rats. In normal, glycated hemoglobin make up 3.4-5.8% of total hemoglobin and a small portion of blood glucose, usually between 4.5-6%, is covalently bonded to the red blood cells in hemoglobin (Kumar et al. 2013c), but the level of glycated hemoglobin was increased in diabetic mellitus patient due to an excess of glucose present in the blood reacts with hemoglobin to form glycated hemoglobin. The level of glycated hemoglobin was increased upto 16% in diabetes mellitus patients (Koeing et al. 1976). Glycated hemoglobin can be used as an indicator of metallic control of diabetes since glycohemoglobin levels approach normal value in diabetes in metabolic control. In this investigation the level of glycated hemoglobin was elevated more than 4 times to the normal control rats. Treatment with different doses of UFD significantly brought back the increased level near normal levels (Table 3), which indicate the improved level of glycemic control. The possible mechanism of action of the UFD in the glycated hemoglobin may be decreasing the blood glucose level and inhibit the addition of the glucose with the hemoglobin.

Hypercholesterolemia and hypertriglyceridemia are mostly found in the diabetes due to lipid abnormalities (Shepherd 2005). These are the major factor involved in rising of coronary heart disease and atherosclerosis, which are the secondary complication accompanying during diabetes (Ananthan et al. 2003). The level of triglyceride increased due to insulin deficiency resultant failure to activate lipoprotein lipase thereby causing hypertriglyceridemia (Shirwaikar et al. 2005). In diabetes, the deposition of the cholesterol in the peripheral tissue is carrying by LDL and VLDL, peripheral tissue to survive and then excretion of cholesterol done by HDL. Hence increased level of LDL and VLDL is atherogenic. The level of serum lipids was elevated 2 times more as compared to the normal control rats. Treatment of different doses of UFD significantly controls the increased level of serum lipids (Triglyceride, Low density lipoprotein, VLDL) and significantly increased the level of HDL in diabetic control rats.

Lately, many investigators have been concentrated on the role of oxidative stress in diabetes. The investigator claims that oxidative stress plays an important role in the development of the diabetic complications (Sepici-Dinçel et al. 2009). SOD, CAT, GPx plays a significant role in preventing the cell damaging from oxidative stress. During the oxidative stress, production of free radical starts, once generated, it continuously react to each other and formed the new free radicals (Kumar et al. 2013c). These free radicals react with all biological substances (mainly polyunsaturated fatty acids) in the body and continuous reaction of the free radical lead to lipid peroxidation. Increased level of lipid peroxidation in the body decrease the membrane fluidity, change the membrane bound receptor and impaired enzyme activity of membrane function (Arulselvan and Subramanian 2007). In our investigation, the level of SOD, CAT, GPx was decreased and the level of MDA (as an indicator of LPO) increased in STZ induced diabetic rats, having high rate of free radical generation. But treatment with different doses of UFD significantly decreased the level of MDA. The decreased in the level of MDA, an increase in the level of GPx was observed, which led to deactivation of LPO reaction. ROS (Reactive oxygen species) directly eliminated by primary enzyme such as SOD and CAT. SOD, is capable of changing the superoxide radical anions (O_2_^-^) into hydrogen peroxide (H_2_O_2_) and CAT is capable to the reduction of hydrogen peroxide and involved in detoxification of hydrogen peroxide (H_2_O_2_) concentration. Some time in diabetes the level of SOD was increased without increasing the level of GPx, that in the cell facing the overload of peroxidases. Then the cell peroxide reacts with the transitional metals and immediately formed the hydroxyl radicals, production of hydroxyl radicals is very harmful to the cells (Halliwell and Gutteridge 1989). STZ induced diabetes inactivate the activated antioxidant enzyme such as SOD, CAT, and GPx by fluctuating these proteins thus producing induced oxidative stress, continuously oxidative stress caused the LPO (Kennedy and Lyons 1997). In our investigation the SOD and CAT significantly decreased the diabetes as a result of non-enzymatic glycosylation and oxidation (Al-Azzawie and Alhamdani 2006). The possible mechanism of action of the UFD may be enhancing the level of the endogenous antioxidant enzymes.

Liver is vital organ that play an important role in defense of the postprandial hyperglycemia and involved in the glucose metabolism (synthesis of glycogen). In liver, glucose is converted into glucose-6-phosphatase by the help of hexokinase (Latha and Pari 2003; Baquer et al. 1998). STZ induced diabetic rats decrease glycolysis, disturb the capacity of the liver to synthesize glycogen and decreased the level of hexokinase. Decreased level of hexokinase showed, an effect on glycolysis and inhibits the utilization of glucose for energy production (Raju et al. 2001). The STZ induced diabetic rats treated with different doses of UFD brought back the activity of this enzyme near to normal control and increases the utilization of glucose for energy conversion. Another liver vital enzyme is glucose-6-phosphatase which regulates the glucose metabolizing enzyme. In STZ induced diabetic rats increased level of glucose-6-phosphatase boost the production of fats from carbohydrates and increased the fats deposition in the liver and kidney (Liu et al. 1994). Some investigators claim that increased level of glucose-6-phosphatase enhanced the activity of a gluconeogenetic enzyme (Bopanna et al. 1997). STZ induced diabetic rats treated with different doses of UFD had brought back the activity of glucose-6-phosphatase enzyme near to normal control. Fructose-1-6-biphosphate is the vital enzyme of the liver plays an important role in the glycolysis, its convert glucose into the energy (Gold 1970). STZ induced diabetic rats increased the level of fructose-1-6-biphosphate. Three different doses of UFD decreased the level of fructose-1-6-biphosphate near the normal control rats.

## Conclusion

Consequently, our research exertion clearly depicts the beneficial effects of umbelliferon-α-D-glucopyranosyl-(2I → 1II)-α-D-glucopyranoside in the STZ induced diabetic rats. Furthermore, the research is in process in our laboratory to explicate the exact mechanism of action of umbelliferon-α-D-glucopyranosyl-(2I → 1II)-α-D-glucopyranoside at molecular level.

## Electronic supplementary material

Additional file 1: Supplementary data. (DOCX 214 KB)

## Authors’ original submitted files for images

Below are the links to the authors’ original submitted files for images. Authors’ original file for figure 1
Authors’ original file for figure 2
Authors’ original file for figure 3
Authors’ original file for figure 4
Authors’ original file for figure 5
Authors’ original file for figure 6
Authors’ original file for figure 7
Authors’ original file for figure 8
Authors’ original file for figure 9
Authors’ original file for figure 10
Authors’ original file for figure 11
Authors’ original file for figure 12
Authors’ original file for figure 13
Authors’ original file for figure 14
Authors’ original file for figure 15
Authors’ original file for figure 16
Authors’ original file for figure 17
Authors’ original file for figure 18
Authors’ original file for figure 19
Authors’ original file for figure 20
Authors’ original file for figure 21
Authors’ original file for figure 22
Authors’ original file for figure 23
Authors’ original file for figure 24
Authors’ original file for figure 25
Authors’ original file for figure 26
