# Supplementary material for: RETRACTED ARTICLE: Enhanced glycemic control, pancreas protective, antioxidant and hepatoprotective effects by umbelliferon-α-D-glucopyranosyl-(2I → 1II)-α-D-glucopyranoside in streptozotocin induced diabetic rats
Source: Springerplus. 2013 Nov 28;2(1):639. doi: 10.1186/2193-1801-2-639 (PMC3862866; doi:10.1186/2193-1801-2-639)

**Supplementary data**

**Enhanced glycemic control, pancreas protective, antioxidant and hepatoprotective effects by umbelliferon-α-D-glucopyranosyl-(2^I^→1^II^)-α-D-glucopyranoside in streptozotocin induced diabetic rats.**

Vikas Kumar^1^*, Mohd. Mujeeb^2^*

^1^Department of Pharmaceutical Sciences, Faculty of Health Sciences, Sam Higginbottom Institute of Agriculture, Technology & Sciences, Allahabad, Uttar Pradesh, India – 211007

^2^Department of Phytochemisty & Pharmacognosy, Faculty of Pharmacy, Jamia Hamdard, New Delhi, India – 110062.

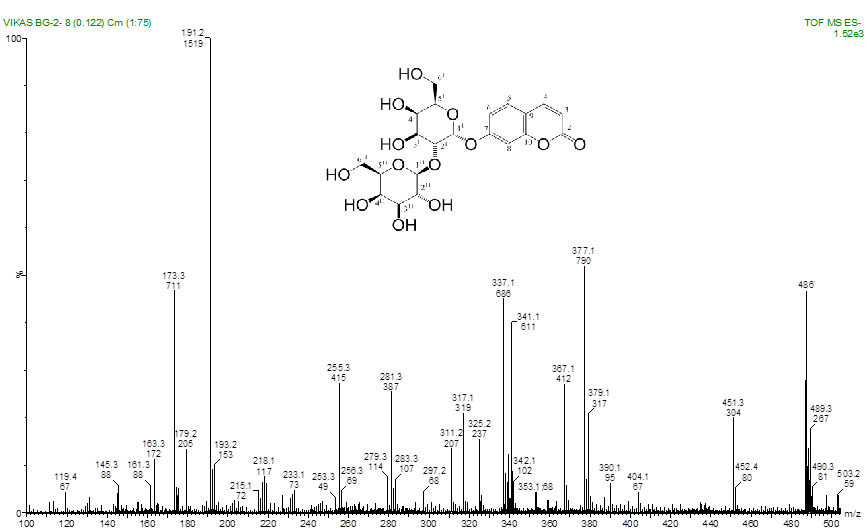

Supplement: Supplementary file 1 — Additional file 1: Supplementary data. (DOCX 214 KB) [file 40064_2013_693_MOESM1_ESM.docx]
