# Supplementary figures and images for: RETRACTED ARTICLE: Enhanced glycemic control, pancreas protective, antioxidant and hepatoprotective effects by umbelliferon-α-D-glucopyranosyl-(2I → 1II)-α-D-glucopyranoside in streptozotocin induced diabetic rats
Source: Springerplus. 2013 Nov 28;2(1):639. doi: 10.1186/2193-1801-2-639 (PMC3862866; doi:10.1186/2193-1801-2-639)

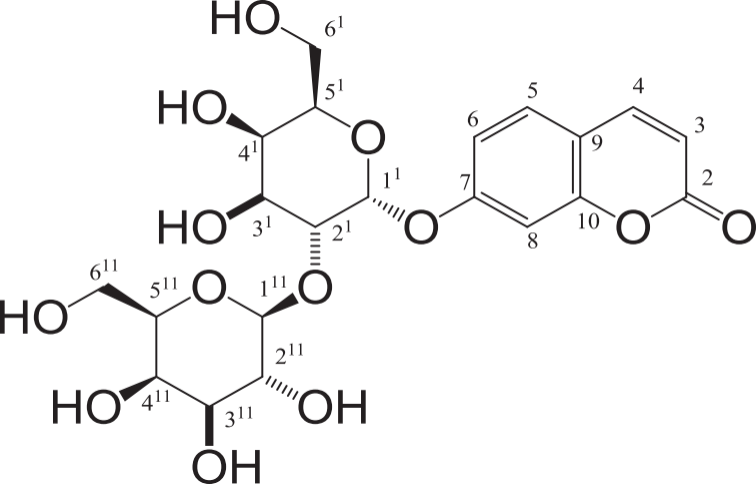

Supplement: Supplementary file 2 — Authors’ original file for figure 1 [file 40064_2013_693_MOESM2_ESM.pdf]

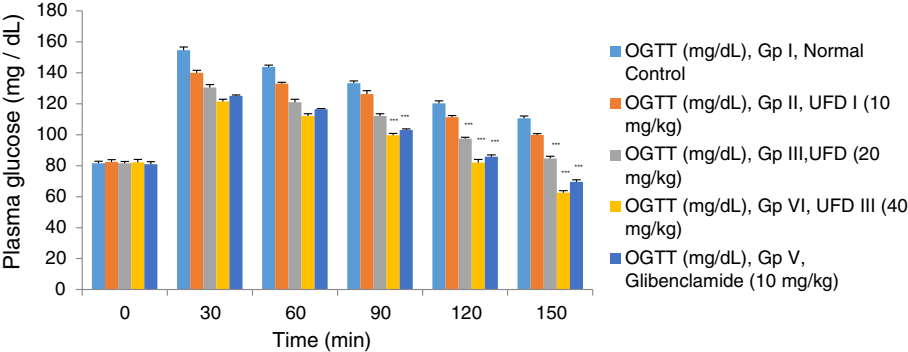

Supplement: Supplementary file 3 — Authors’ original file for figure 2 [file 40064_2013_693_MOESM3_ESM.pdf]

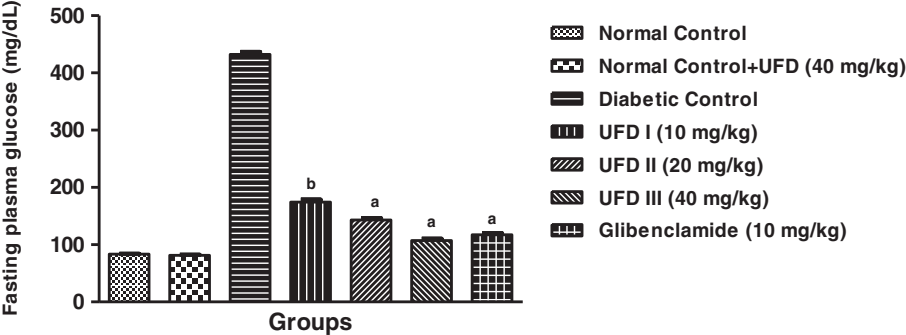

Supplement: Supplementary file 4 — Authors’ original file for figure 3 [file 40064_2013_693_MOESM4_ESM.pdf]

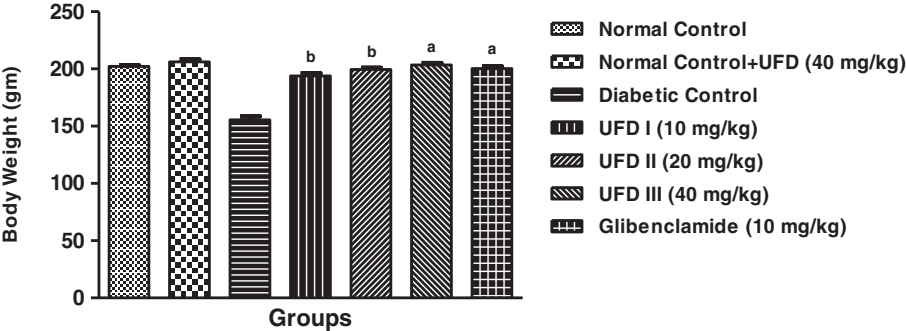

Supplement: Supplementary file 5 — Authors’ original file for figure 4 [file 40064_2013_693_MOESM5_ESM.pdf]

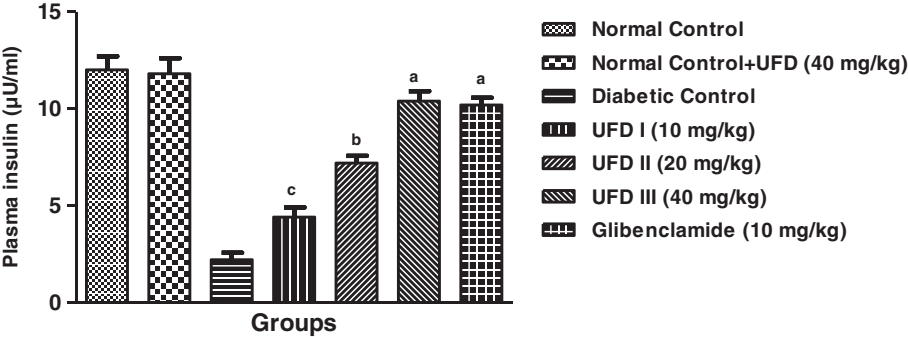

Supplement: Supplementary file 6 — Authors’ original file for figure 5 [file 40064_2013_693_MOESM6_ESM.pdf]

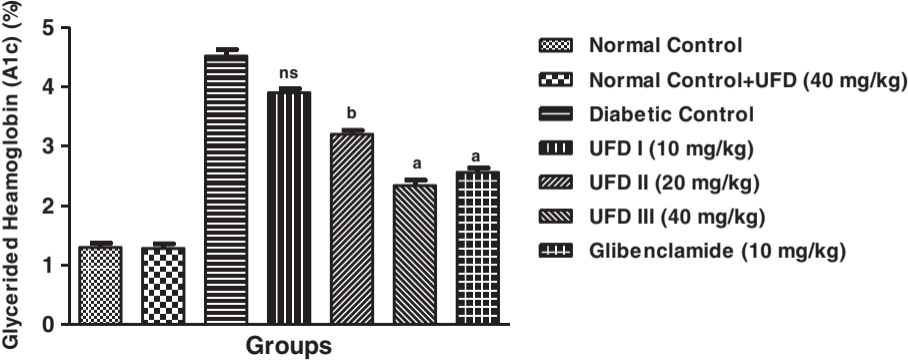

Supplement: Supplementary file 7 — Authors’ original file for figure 6 [file 40064_2013_693_MOESM7_ESM.pdf]

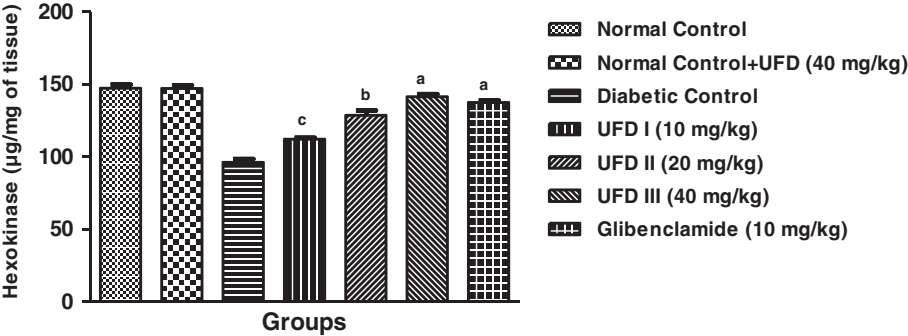

Supplement: Supplementary file 8 — Authors’ original file for figure 7 [file 40064_2013_693_MOESM8_ESM.pdf]

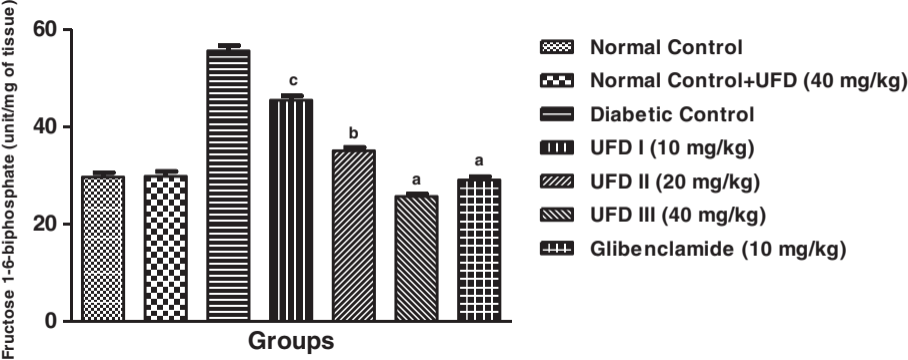

Supplement: Supplementary file 10 — Authors’ original file for figure 9 [file 40064_2013_693_MOESM10_ESM.pdf]

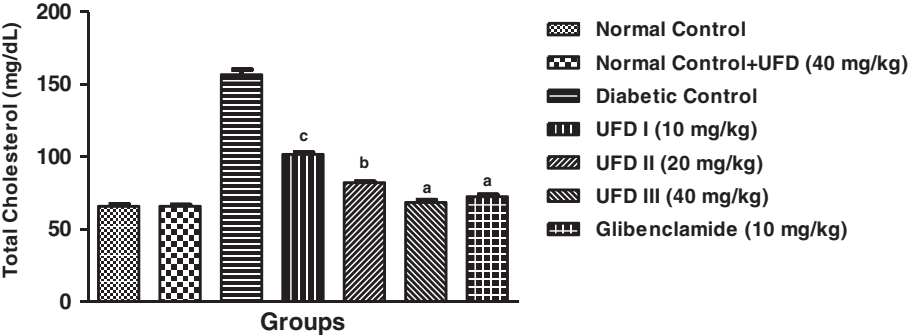

Supplement: Supplementary file 11 — Authors’ original file for figure 10 [file 40064_2013_693_MOESM11_ESM.pdf]

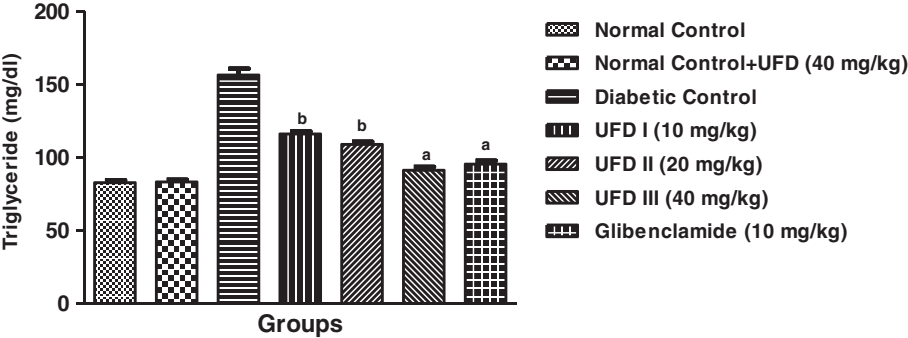

Supplement: Supplementary file 12 — Authors’ original file for figure 11 [file 40064_2013_693_MOESM12_ESM.pdf]

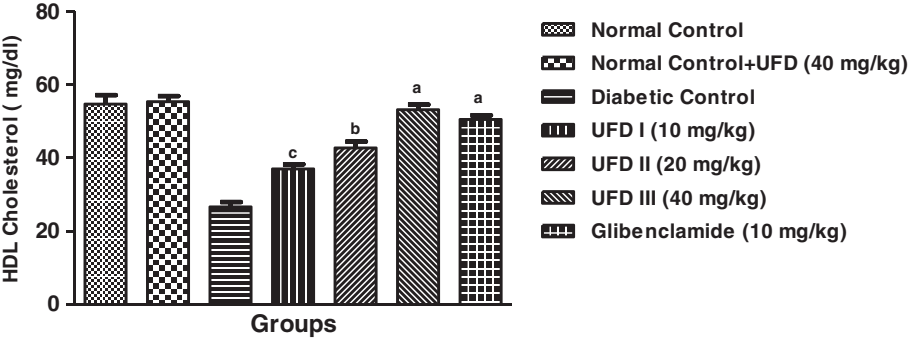

Supplement: Supplementary file 13 — Authors’ original file for figure 12 [file 40064_2013_693_MOESM13_ESM.pdf]

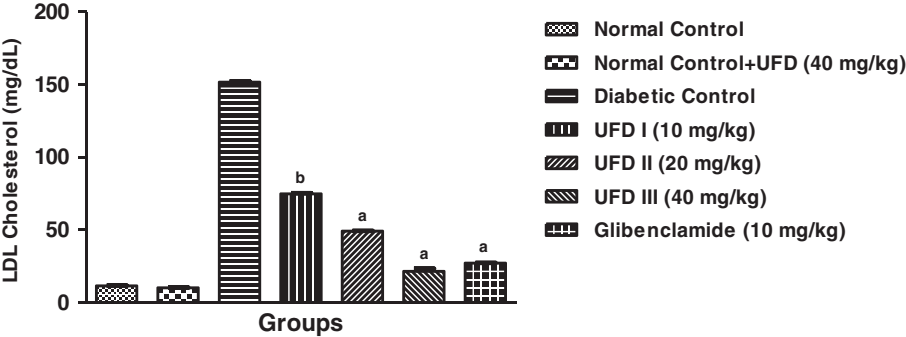

Supplement: Supplementary file 14 — Authors’ original file for figure 13 [file 40064_2013_693_MOESM14_ESM.pdf]

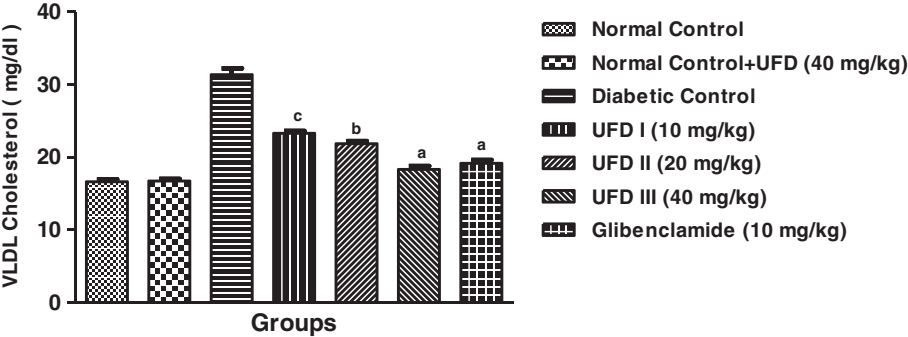

Supplement: Supplementary file 15 — Authors’ original file for figure 14 [file 40064_2013_693_MOESM15_ESM.pdf]

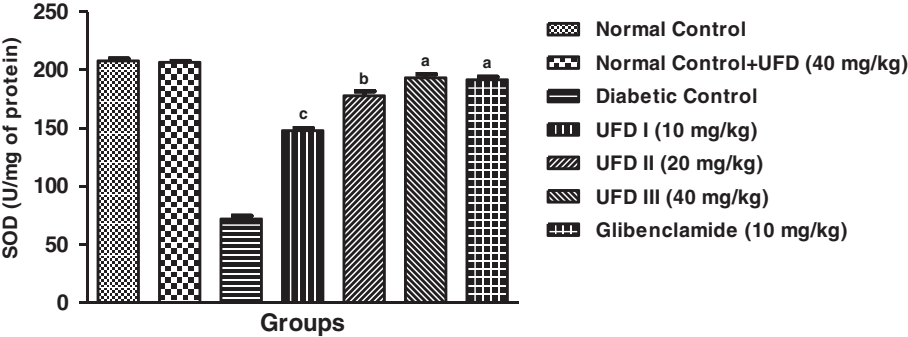

Supplement: Supplementary file 16 — Authors’ original file for figure 15 [file 40064_2013_693_MOESM16_ESM.pdf]

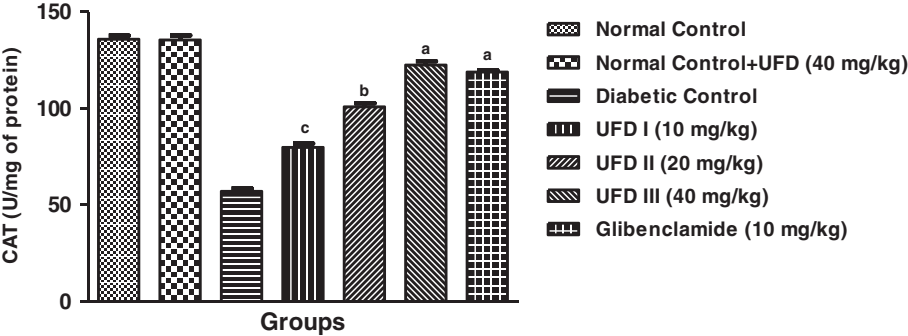

Supplement: Supplementary file 17 — Authors’ original file for figure 16 [file 40064_2013_693_MOESM17_ESM.pdf]

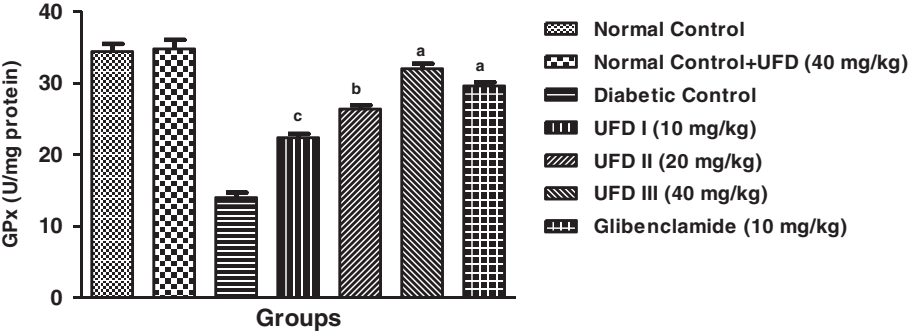

Supplement: Supplementary file 18 — Authors’ original file for figure 17 [file 40064_2013_693_MOESM18_ESM.pdf]

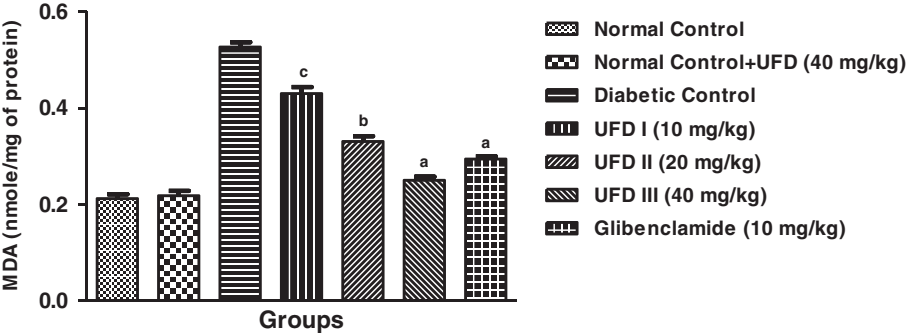

Supplement: Supplementary file 19 — Authors’ original file for figure 18 [file 40064_2013_693_MOESM19_ESM.pdf]

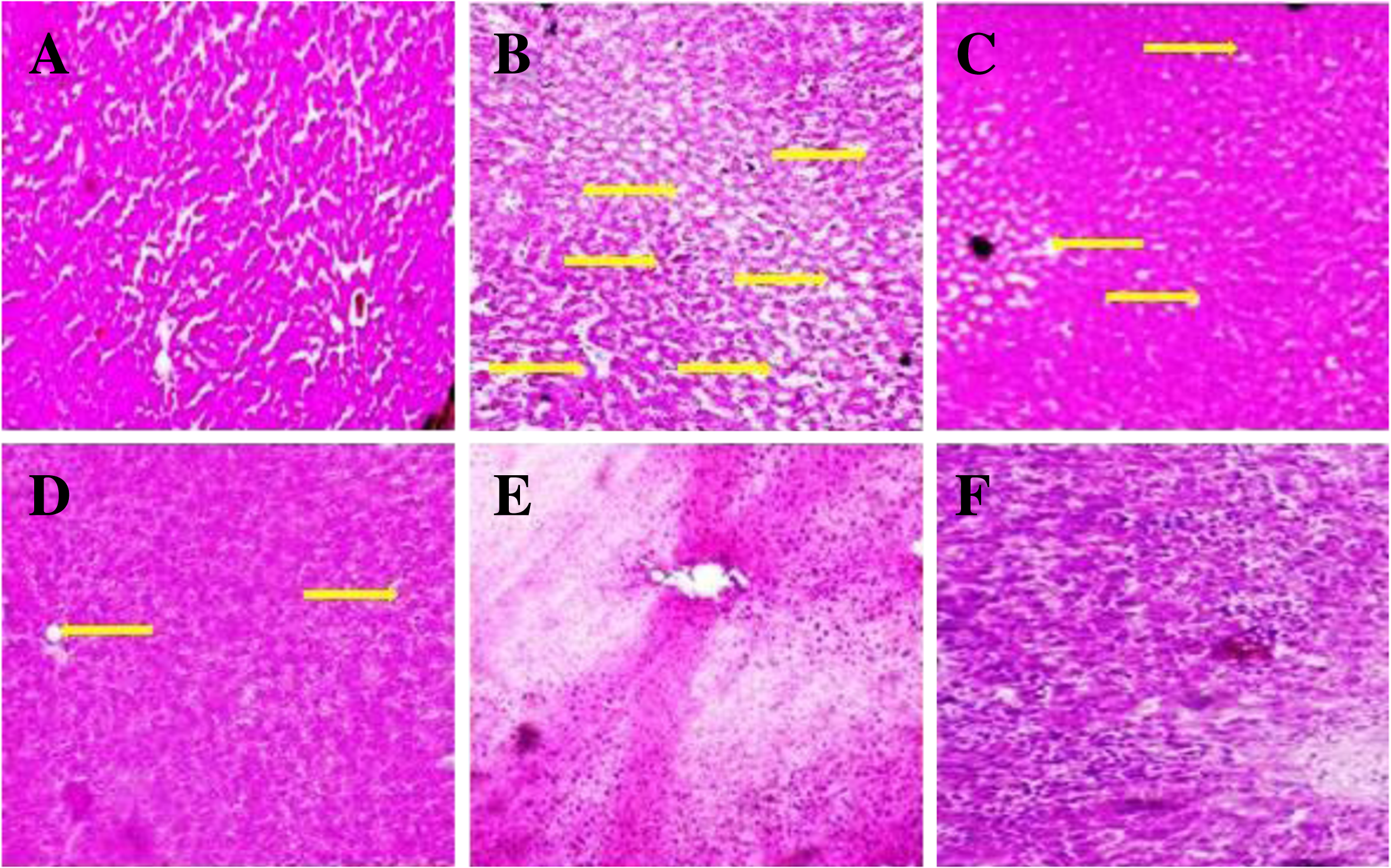

Supplement: Supplementary file 20 — Authors’ original file for figure 19 [file 40064_2013_693_MOESM20_ESM.tif]

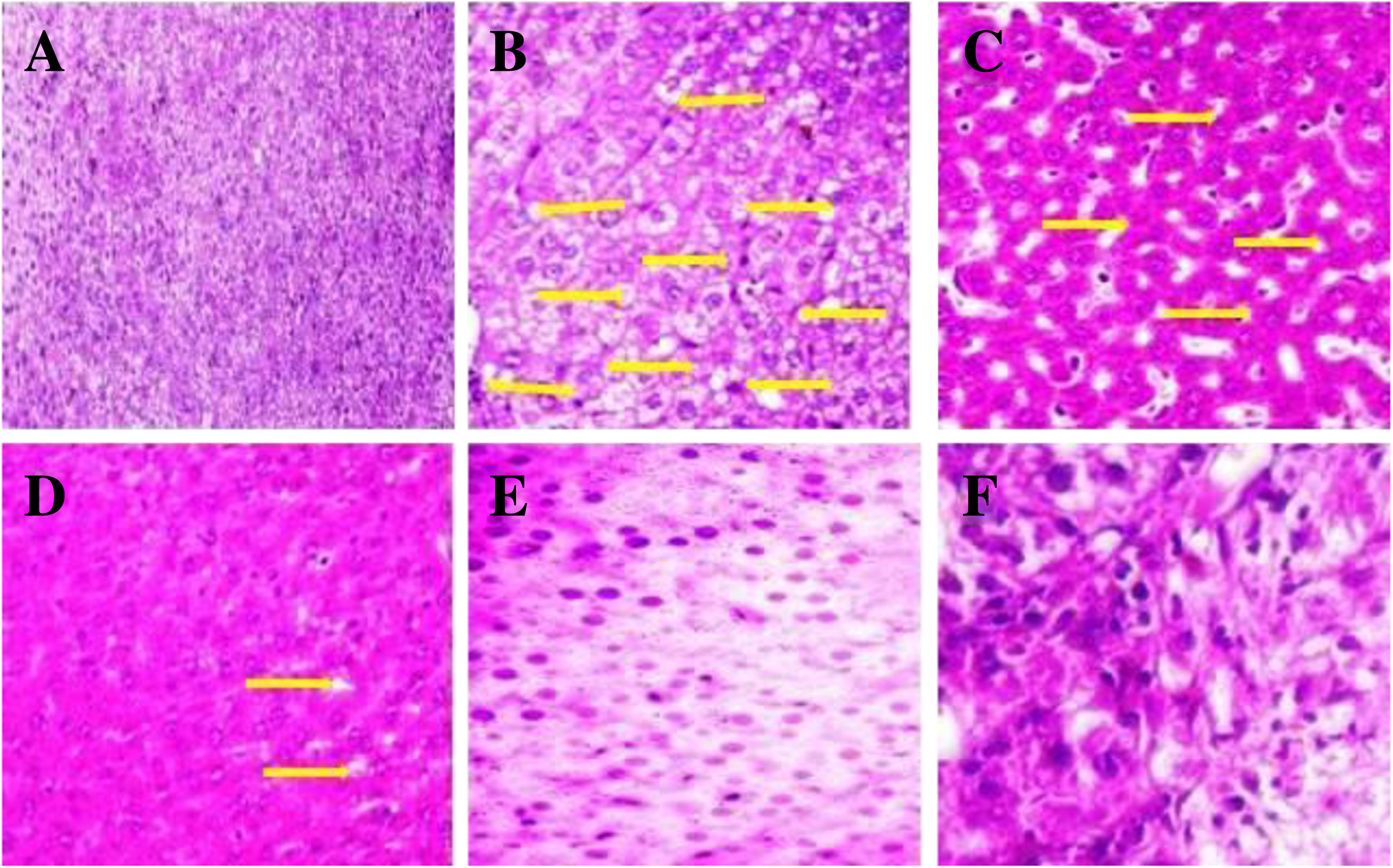

Supplement: Supplementary file 21 — Authors’ original file for figure 20 [file 40064_2013_693_MOESM21_ESM.tif]

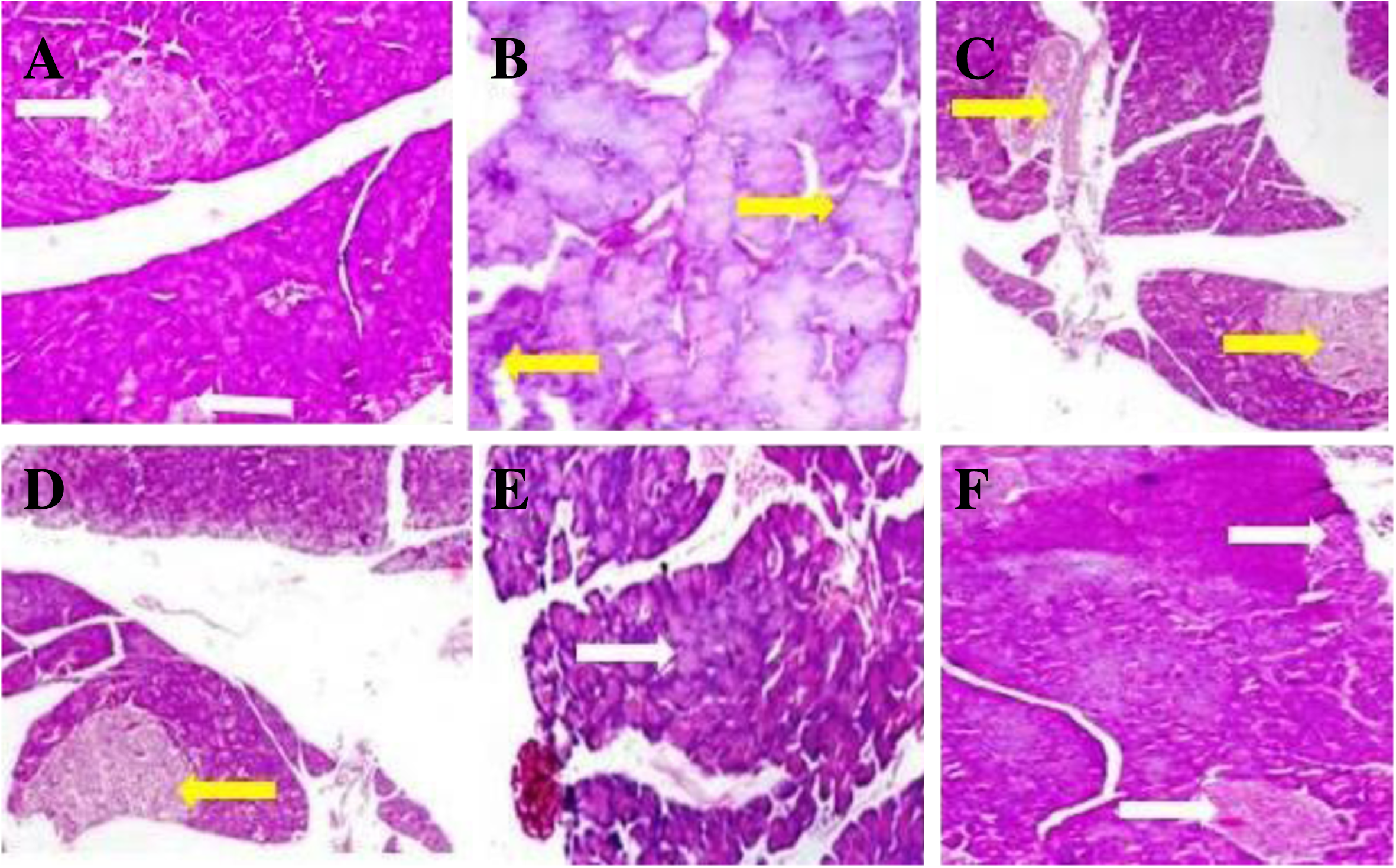

Supplement: Supplementary file 22 — Authors’ original file for figure 21 [file 40064_2013_693_MOESM22_ESM.tiff]

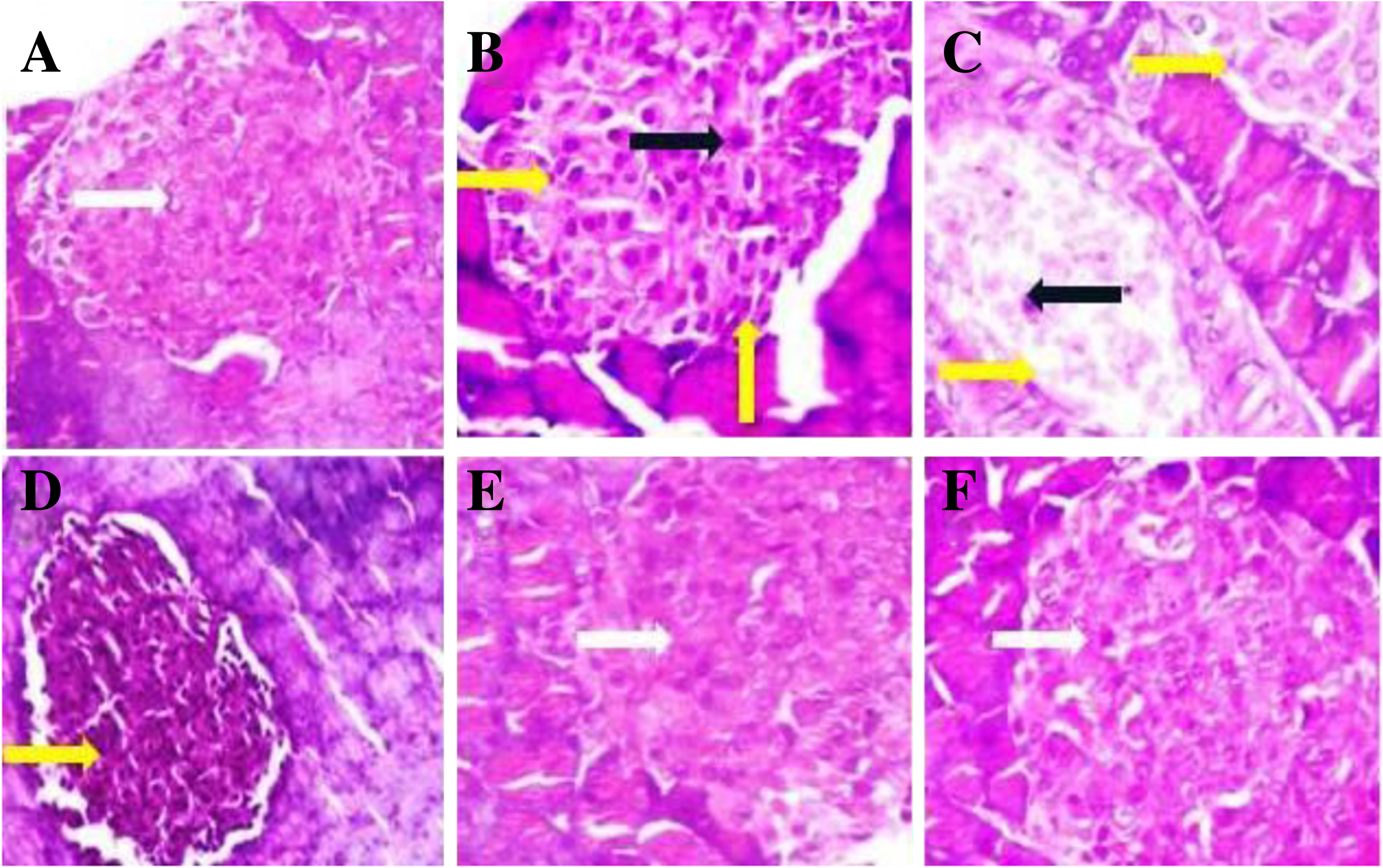

Supplement: Supplementary file 23 — Authors’ original file for figure 22 [file 40064_2013_693_MOESM23_ESM.tiff]

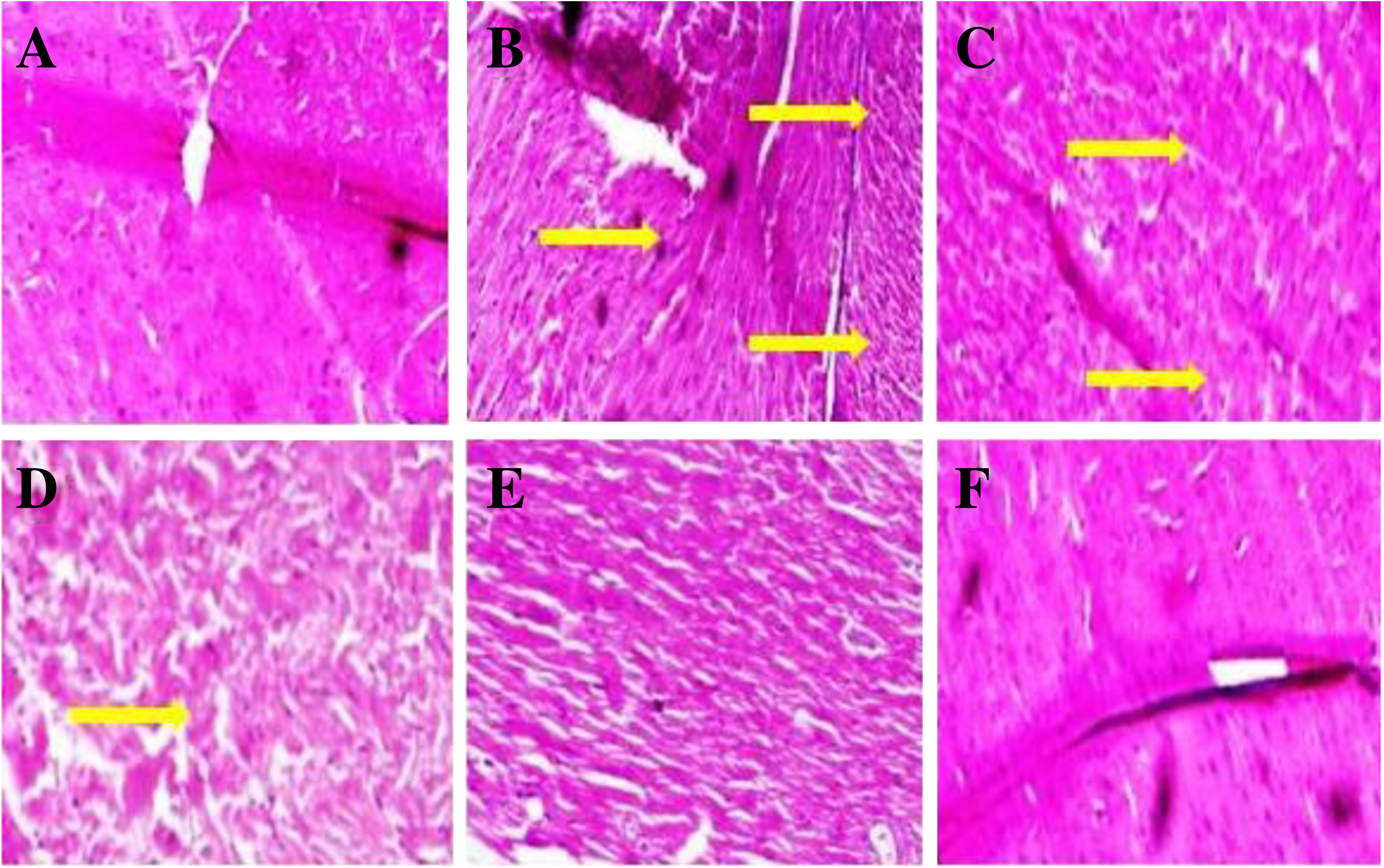

Supplement: Supplementary file 24 — Authors’ original file for figure 23 [file 40064_2013_693_MOESM24_ESM.tiff]

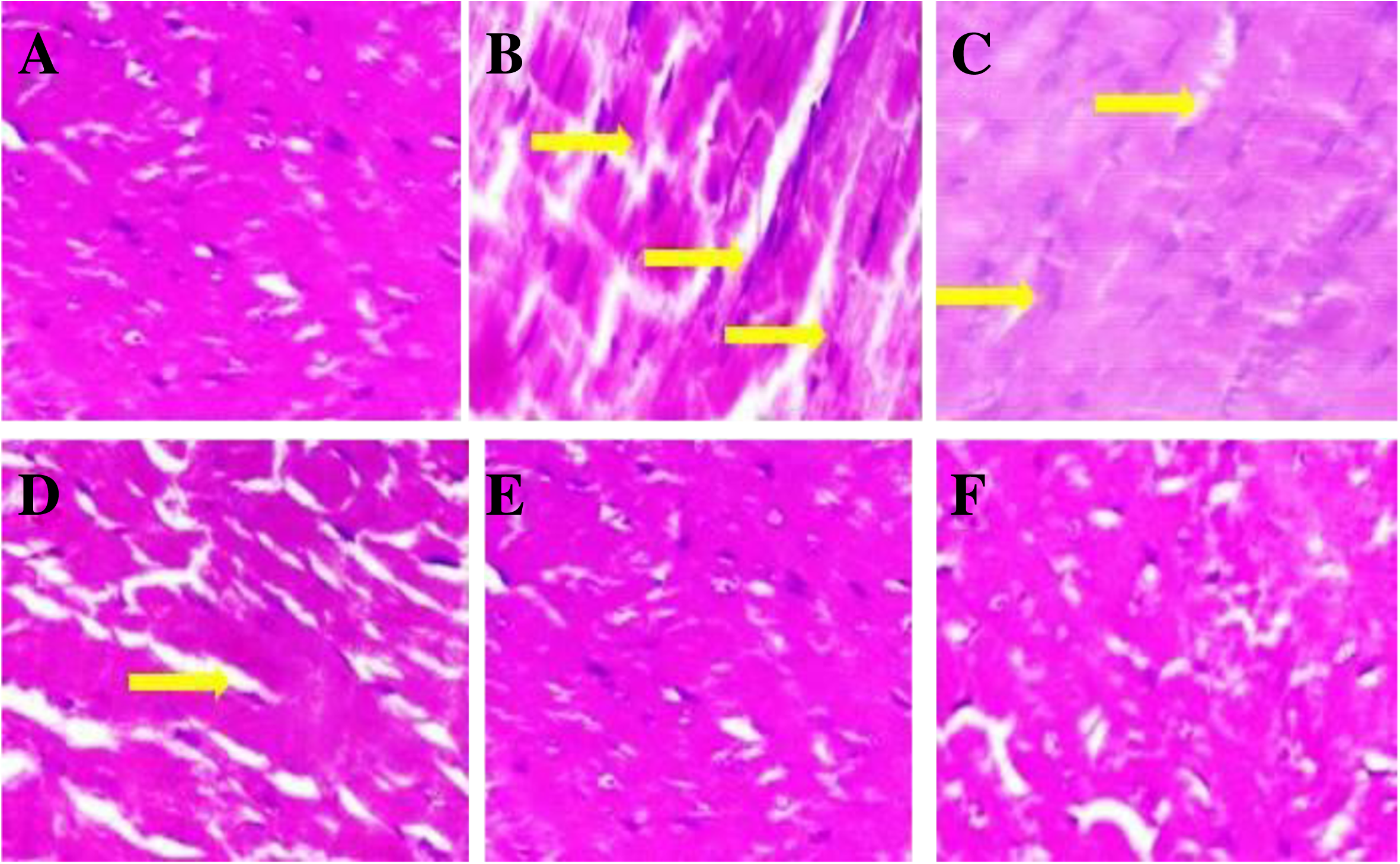

Supplement: Supplementary file 25 — Authors’ original file for figure 24 [file 40064_2013_693_MOESM25_ESM.tiff]

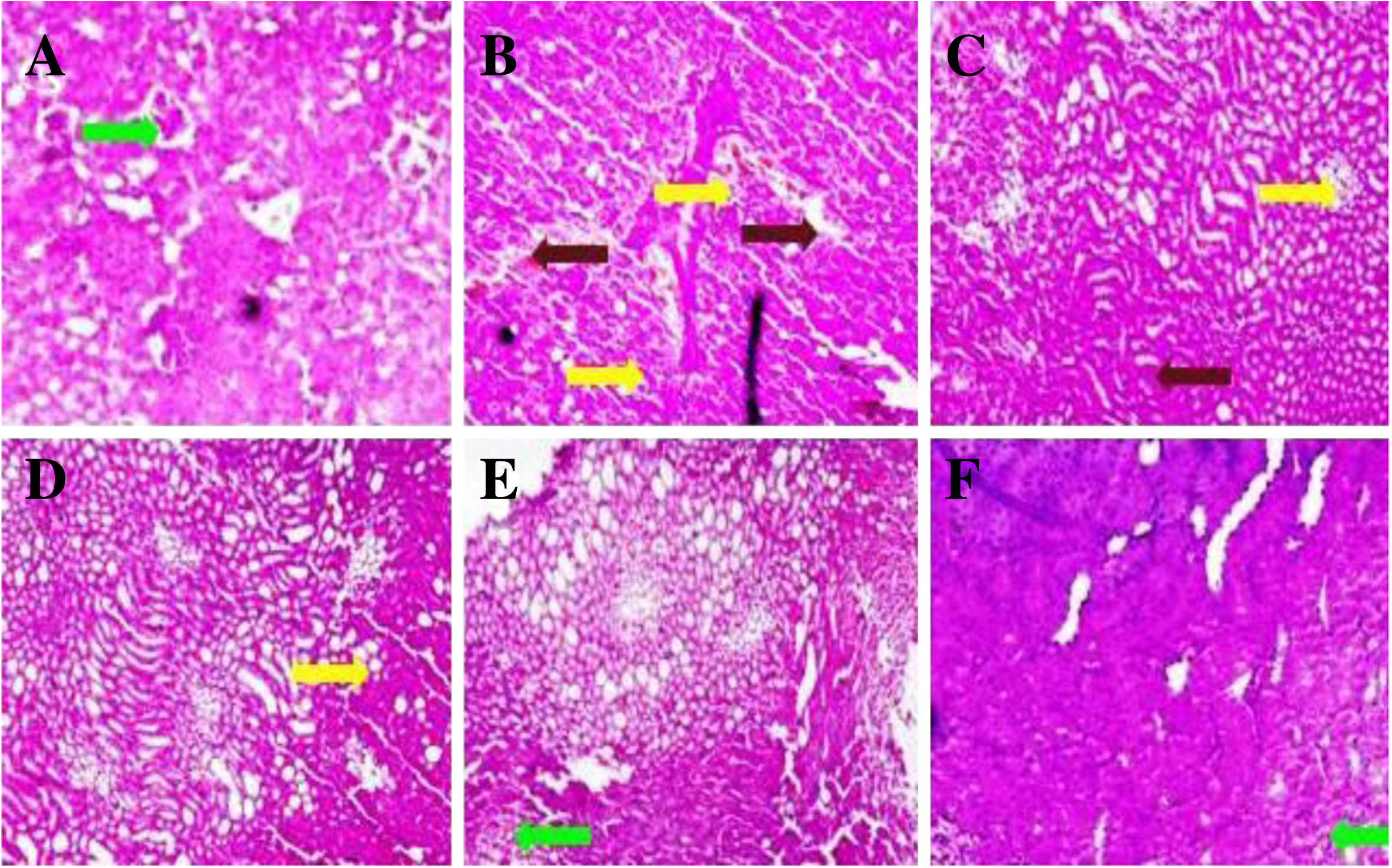

Supplement: Supplementary file 26 — Authors’ original file for figure 25 [file 40064_2013_693_MOESM26_ESM.tiff]

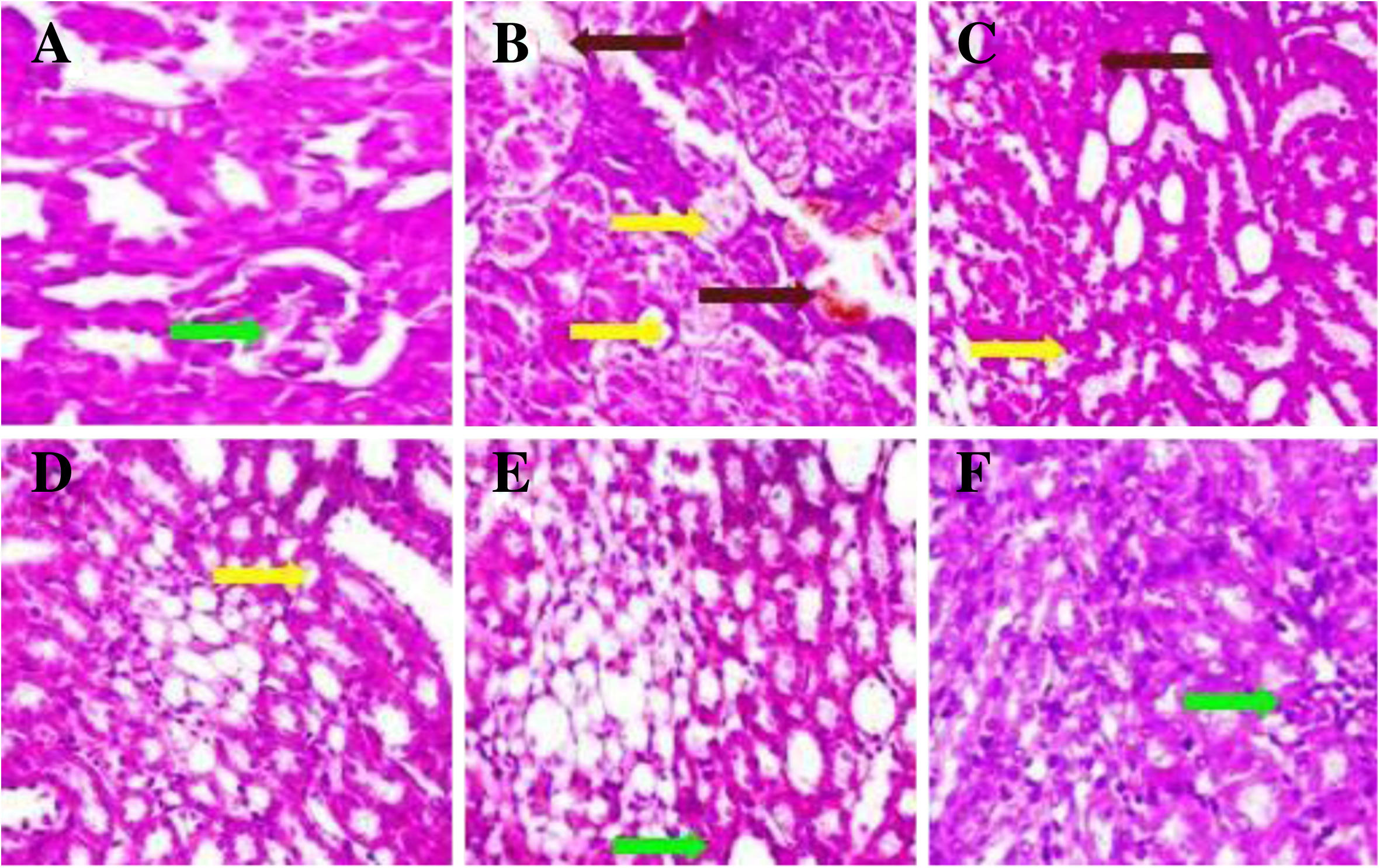

Supplement: Supplementary file 27 — Authors’ original file for figure 26 [file 40064_2013_693_MOESM27_ESM.tiff]
