# Supplementary material for: RETRACTED ARTICLE: Enhanced glycemic control, pancreas protective, antioxidant and hepatoprotective effects by umbelliferon-α-D-glucopyranosyl-(2I → 1II)-α-D-glucopyranoside in streptozotocin induced diabetic rats
Source: Springerplus. 2013 Nov 28;2(1):639. doi: 10.1186/2193-1801-2-639 (PMC3862866; doi:10.1186/2193-1801-2-639)
Supplement: Supplementary file 9 — Authors’ original file for figure 8 [file 40064_2013_693_MOESM9_ESM.pdf]

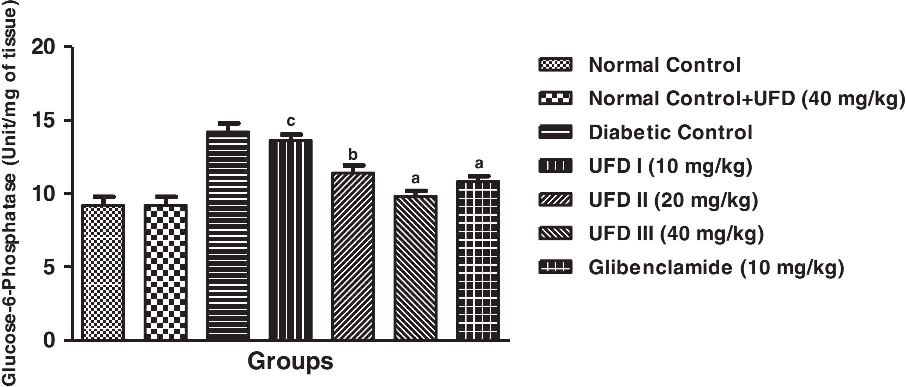

Glucose-6-Phosphatase (Unit/mg of tissue)
20
15
10
5
0
c
b
a
a
Groups
Normal Control
Normal Control+UFD (40 mg/kg)
Diabetic Control
UFD I (10 mg/kg)
UFD II (20 mg/kg)
UFD III (40 mg/kg)
Glibenclamide (10 mg/kg)
